# Supplementary material for: Summer‐born struggle: The effect of school starting age on health, education, and work
Source: Health Econ. 2020 Feb 12;29(5):591–607. doi: 10.1002/hec.4005 (PMC9328428; doi:10.1002/hec.4005)
Supplement: Supplementary file 1 — Table 1: Descriptive Statistics Table 2: Reduced‐Form Effect of SSA on Special Needs Table 3: Reduced‐Form Effect of SSA on Educational Outcomes Table 4: Reduced‐Form Effect of SSA on Post‐Compulsory Education Choice Figure 1: The Effect of SSA on Labor Market Outcomes Figure 2: Graphical First Stage Table A1: Alternative Specification for the SSA Effect on Post‐Compulsory Education [file HEC-29-591-s001.pdf]

Summer-Born Struggle: The Effect of School Starting Age on Health, Education, and Work*

Simone Balestra[1], Beatrix Eugster[1], and Helge Liebert[2]

[1] *University of St. Gallen, Switzerland*
[2] *Harvard University, USA*

Abstract

Children starting school older consistently exhibit better educational outcomes. In this paper, we underscore child development as a mechanism driving this effect. Using unique administrative data on health examinations, we study the causal effect of school starting age (SSA) on a child's probability of being diagnosed with special educational needs in early grades. Results show that children with higher SSA are less likely to develop behavioral problems and speech impediments, whereas learning disabilities, ADHD, and dyslexia/dyscalculia remain unaffected. Importantly, these effects only arise after primary school entry and are not due to pre-existing health conditions. We also find that teachers tend to over-refer relatively younger children to special needs services, but this over-referring behavior is not driving the results, which are based on psychologists' diagnoses. The SSA-effect persists throughout compulsory schooling, resulting in higher test scores and better-quality vocational training contracts. However, SSA does not affect employment, earnings, or disability insurance benefits at labor market entry.

Keywords: school starting age, special needs, school performance.

JEL Classification: I14, I21, J13.

* We are grateful to the editor and two anonymous referees for helpful comments. The paper has benefited from constructive comments by Christina Felfe, Mathias Huebener, Michael Lechner, Nicole Maestas, Marc-Andreas Muendler, Hannes Schwandt, Anthony Strittmatter, and Stefan Wolter. For the data, we thank the School Psychological Service, the Ministry of Education of the canton of St. Gallen, the Stellwerk test service provider, the Swiss Federal Statistical Office, and the Swiss Central Compensation Office. The paper benefited from valuable feedback at the following meetings: European Association of Labor Economists, Ski and Labor seminar, Swiss Society of Economics and Statistics, and departmental seminars at the universities of Groningen, St. Gallen, Wuppertal, and Zurich. We acknowledge financing from the Swiss National Science Foundation (grant no. 176381) and declare no conflict of interest. This study has been approved by the Ethics Committee of the University of St. Gallen. Corresponding author: Beatrix Eugster, University of St. Gallen, Rosenbergstrasse 51, CH-9000 St. Gallen, beatrix.eugster@unisg.ch, +41 71 224 31 79.

1 Introduction

Virtually all education systems have a single cutoff date that determines when children become eligible for compulsory schooling. This cutoff rule creates a continuum of ages at school entry, whereby the oldest child is up to one year older than his or her youngest classmates. Research has shown that age of school entry is an important determinant of early student achievement (Black, Devereux, and Salvanes, 2011; Elder and Lubotsky, 2009; Fredriksson and Öckert, 2014). Although this pattern is consistent across countries (Bedard and Dhuey, 2006), the underlying mechanism that supports this empirical regularity and the long-term effects of school starting age remains unclear.

A prominent explanation from developmental psychology is maturity (Whitebread, 2012). While children are ready to learn at all ages, young children are usually less prepared to engage in academic work than their older peers (Morrison, Alberts, and Griffith, 1997; Stipek and Ryan, 1997) and more vulnerable to external influences (Datar and Gottfried, 2015). This developmental disadvantage might trigger special educational needs in children, for instance due to increased incidence of learning impairments or behavioral problems.[1] Individuals who develop special needs during childhood have a higher risk of subsequent history of unsuccessful education, difficulties in labor market integration, and lower earnings during adulthood (Hanushek, Kain, and Rivkin, 2002; Wagner and Blackorby, 1996). In addition, educating children with special needs is considerably more costly than educating children without special needs (Duncombe and Yinger, 2005). It is thus imperative for policy makers to understand the role of school starting age (SSA) in explaining child development, with respect to not only achievement but also to special needs demand.

In this paper, we study the causal effect of SSA on a child's probability of being diagnosed with special educational needs in early grades. We then assess the persistence of SSA effects in terms of scholastic achievement at the end of compulsory schooling and on labor market entry. While the literature on the effects of SSA is extensive, it remains unsettled which individual differences are triggered by SSA and when they emerge. The aim of the present paper is to fill this research gap. In particular, we offer the most comprehensive analysis to date of the incidence, onset, and type of special needs conditions induced by SSA by utilizing detailed data on medical examinations. In addition, we jointly analyze educational and labor market outcomes above and beyond those previously examined in the literature within the same research design. We are able to credibly identify the effects of SSA through a regression discontinuity design based on the exact day of birth. In Switzerland, children enter compulsory education[2] in the fall if they have reached age four before August 1 of the same year. This institutional rule allows us to compare children born around the cutoff, children who are observationally similar but enter school at different ages.

We base our analyses on the complete school cohorts in the Swiss canton of St. Gallen born from 1992

[1] According to ICD-10 diagnosis guidelines, special needs is an umbrella term for special educational requirements resulting from learning disabilities, communication disorders, emotional and behavioral disorders, physical disabilities, and developmental disabilities.

[2] In Switzerland, compulsory education consists of two years of kindergarten, six years of primary school, and three years of secondary school.

to 2003.[3] For these children we have administrative information on special needs diagnoses from the School Psychological Service. Additionally, we observe test scores in Math and German in grade eight, when students take a compulsory standardized exam. We further merge the data with administrative records on both vocational education and training (VET) and high schools (academic track). Finally, we link our data to administrative records on earnings and employment from the Central Compensation Office, the social security administration in Switzerland. The high-quality administrative data allow to investigate the differential effects of SSA over children's educational trajectories in great detail.

Prior research shows some evidence that increases in SSA reduce children's risk of disability classification (Dhuey and Lipscomb, 2010; Elder, 2010) and improve measures of mental health (Dee and Sievertsen, 2018; Mühlenweg et al., 2012). We contribute to this literature along three dimensions. First, we present novel evidence on the relationship between SSA and special needs diagnosis. Our data and research design allow to pinpoint exactly how SSA mediates through special needs and how its effects perpetuate through young adults' educational career into the labor market. Moreover, with respect to reliability and wealth of information, our data constitute a major improvement on existing data sets, which are primarily retrospective non-expert surveys. By relying on diagnoses made by medical professionals, we highlight which conditions are triggered by differences in SSA and which are unaffected.

Second, we shed light on the interplay between experts' evaluation and educators' behavior towards special education classification. Dhuey and Lipscomb (2010) and Schwandt and Wuppermann (2016) show that, in some cases, special needs classification (or ADHD misdiagnosis) is utilized as a supplemental service that targets additional resources at younger students. In the presence of this over-referring, experts' evaluations of special needs become crucial. When specialists – as compared to teachers or parents – perform the diagnoses, the risk of applying relative standards is much smaller (Dalsgaard et al., 2012). We deal with this issue by distinguishing between the decision to refer a child for special needs evaluation (made usually by teachers) and the results of an expert evaluation, which is made by SPS professionals. While the existing studies can only speculate on the role of educators in the increase of SN referrals among young children, we directly tackle this issue because in our setting the teacher decides whether to refer a child or not.

Third, the data allow us to perform a comprehensive assessment of the medium- and long-run effects of SSA within a uniform institutional framework. The current literature on the long-term effects of SSA is mixed, finding small or zero effects on employment and earnings (Dustmann, Puhani, and Schönberg, 2017; Fredriksson and Öckert, 2014). In the medium run, we examine the effect of SSA on grade retention and test scores at the end of compulsory education. Moving further towards long-run persistence, we estimate the effects on post-compulsory education trajectories and we pose the question of whether SSA effects still matter at the age of labor market entry and investigate employment, earnings, and disability in the early years after compulsory schooling. The education-health gradient is a key parameter in models of the demand for health

[3]Switzerland is a federal republic comprising 26 member states called cantons. St. Gallen is the fifth largest canton in Switzerland with a population of about 500,000.

capital (Grossman, 1972) and models of the influence of childhood development on adult outcomes (Conti, Heckman, and Urzua, 2010). If the health effects of education are large enough, then education policies such as SSA thresholds might become powerful tools for improving health over the life cycle.

The results indicate that children entering school at a younger age have a higher risk of developing special needs than children starting school one year later. Being born shortly before the cutoff date increases the probability of receiving a special needs diagnosis by five percentage points or about 14%. Importantly, this effect is entirely driven by special needs detected after school entry, not due to pre-existing health conditions. By distinguishing by type of special needs, we find that entering school at a younger age mostly increases behavioral problems and speech impediments. The incidence of learning disabilities, dyslexia/dyscalculia, and ADHD are unaffected by SSA. We also show that teachers tend to over-refer younger children for special needs examination. While over-referring occurs for all children, those who were born immediately before the cutoff have a 30% higher probability of being dismissed by the SPS after an initial examination without a diagnosis.

At the end of compulsory education, differences in SSA still affect students' school outcomes. We find that younger students perform worse on standardized tests. While no effects are found for grade repetition during primary and secondary school, we find that younger students are more likely to be allocated to a bridge year between kindergarten and primary school. These results suggest that many children starting school at a younger age will eventually prolong their compulsory education by one year. The differences at the end of compulsory schooling disappear in the long run, when adolescents choose their post-compulsory education track. Children with lower SSA are as likely to both enter the academic track or find an apprenticeship position as their peers with higher SSA. Nonetheless, we find that children starting school younger are significantly less likely to enter the high-quality vocational track. For early labor market outcomes, the data reveal no effect of SSA on earnings and employment for the first seven years after compulsory schooling. Similarly, we find no impact of SSA on the probability of receiving disability insurance benefits.

While these reduced form estimates are informative, it is important to acknowledge that the regression discontinuity design is fuzzy because of individual decisions to delay school start. We estimate local average treatment effects by applying two-sample instrumental variable estimation, because we do not observe actual school starting age for the children in our main data. To construct the first stage, we use the official education statistics for the cohorts born between 2006 and 2011. Results suggest that redshirting is a common practice in Switzerland, but only for children born in June or July, that is, shortly before the cutoff. As expected, the two-sample IV estimations are somewhat larger in size and less precisely estimated than our reduced-form estimates, but overall they confirm our previous results.

The present study provides important insights for policies that can alleviate negative repercussions arising from the discontinuous nature of school starting date cutoffs. The main results indicate which conditions specifically are induced by differential SSA, allowing better targeting of screening policies and remedial actions. Moreover, the results and the institutional context highlight the importance of institutional arrangements that allow for flexible adjustments to children's grade progression during compulsory schooling. Relying on

independent external evaluators, information sharing arrangements and a formal institutionalized process to screen children helps provide equitable access to remedial policies like redshirting or bridge years.

2 Data and Institutional Background

In St. Gallen, children enter compulsory schooling in the fall if they have reached age four before August 1 of the same year. The typical school curriculum consists of two years of kindergarten, six years of primary school, and three years of secondary school. Ability tracking occurs after primary school, with children entering either a higher-ranked (*Sekundarschule*) or a lower-ranked (*Realschule*) track of secondary school. After finishing secondary school, children typically enter either an academic preparation track (i.e., high school) or vocational education.

Inclusive classroom education is a common practice in Swiss schools. Inclusive education (or *mainstreaming*) refers to a model wherein students with special needs spend most or all of their time with non-special needs students. Only about 2% of children – those who have severe physical or mental handicaps which would not allow them to follow a regular curriculum – are educated in special education institutions. We do not have any information about these children in our data. The regular schools, however, offer institutionalized support services for children who develop special educational needs. Special educational needs result from diagnoses associated either with behavioral problems or learning impairments (for example, ADHD, dyslexia/dyscalculia, or speech impediments). In such cases, the teacher notifies both the School Psychological Service (SPS) and the parents.[4] The SPS is a centralized service provider for all schools in the canton. Once a child is referred to the SPS, the SPS initiates contact with the family and schedules an assessment meeting. During this meeting, the SPS staff performs a diagnostic evaluation, provides a diagnosis, and recommends therapy if necessary.

Our analysis focuses on the student population of St. Gallen. We combine different data sources to assemble educational histories for all school cohorts born from 1992 to 2003. These sources include data on special needs from the SPS, on educational achievement from standardized tests, on post-compulsory education, and register data on employment and earnings. We observe the exact date of birth, gender, and whether a pupil is a native German speaker for the entire population of students.

For every child who has ever been in contact with the SPS, we observe the age at registration and reason for registration (for example, learning difficulties, disruptive behavior, or family problems), the number of consultations, and comprehensive information on the diagnoses and the suggested treatments. This data set allows us to construct measures of the onset and severity of special needs. Furthermore, we can distinguish between non-expert and expert assessment by comparing the teacher-initiated registration at the SPS with the SPS staff assessment.

The achievement data are based on a compulsory standardized test taken in eighth grade, "Stellwerk 8."

[4]Children are 90% of the times referred to the SPS by the teacher, whereas the remaining registering parties are either the parents (6%) or various civil authorities (4%). For the analysis, we consider all referrals regardless of the registering party, in order to avoid differential selection on either side of the cutoff. The results remain unchanged if we remove non-teacher referrals from the sample.

Stellwerk 8 is a norm-referenced, self-scoring, adaptive, computer-based exam. All students in grade eight – except those enrolled in special education schools – are tested. The test is administered between February and April, towards the end of the school year. The test results are important for students. After the test, students receive a certificate with their results. This certificate is usually provided to potential employers when students apply for apprenticeship positions during ninth grade, the last year of compulsory education.

In addition to the data on school performance, we add data about students' career path after compulsory education. Upon finishing compulsory schooling, most Swiss students enter VET by applying for VET positions and signing a training contract with a firm.[5] We link our data to information about all VET contracts signed during 2008-2016. A smaller percentage of each cohort enters the academic preparation track to obtain a higher education entrance qualification. We link the children in our data to the administrative high school records to track those who enter the academic track after compulsory schooling. We classify individuals who we do not find in any post-compulsory education register as not pursuing any post-compulsory education, excluding those who are too young to enter.

The last piece of information we add to our data set is information about labor supply and earnings. Workers in Switzerland pay social security taxes on their earnings, and the Swiss Central Compensation Office tracks monthly employment, earnings and social security contributions for all registered workers. We add this information to our data. For individuals in our data who ever worked between 2007 and 2016, we observe how many months per year they worked and their respective (net) earnings.[6]

Finally, we add administrative information on disability insurance (DI) for the year 2016. In detail, of the 52,082 individuals in our main data set, 28,561 (54.8%) paid contributions to their first pillar social security in 2016.[7] Contributions to first pillar accounts are mandatory from age 17 for workers and 20 for everyone, which explains the missing information of almost half of the sample, because many students in our sample are too young to be paying first-pillar contributions. We gained access to the personal accounts of the 28,561 individuals and we observe whether an individual is a DI recipient.

Table 1 shows the descriptive statistics for our sample of 52,082 individuals. About 32% of children are referred to special needs services at some point during their school career. However, 14% of the referred children (4.4% overall) are dismissed without a diagnosis after the initial screening and do not receive further support. The remaining referred children are diagnosed primarily with learning impairments (20% of all children) and behavioral problems (7% of all children). While special educational needs comprise a variety of learning disabilities (for example, dyslexia or dyscalculia), behavioral problems consist of disruptive behavior almost exclusively. These proportions match the incidence reported in aggregate statistics at the federal level

[5] Students who attend a VET program study part-time at school for 1 to 1.5 weekdays. For the remaining time (3.5 to 4 weekdays), students work as apprentices in host companies with whom they have an employment contract for their entire two- to four-year training period. See Oswald and Backes-Gellner (2014) for an overview of the Swiss VET system. For a broader perspective on VET across countries, see Wolter and Ryan (2011).

[6] Matching across data sources is performed on the basis of three elements combined: first name, family name, and date of birth. The probabilistic matching success rate was above 98%.

[7] The first pillar covers old-age and survivors' insurance, disability insurance, and compensation for loss of earnings (due to maternity leave or military service).

and are also in line with figures reported from other OECD countries (OECD, 2008). While the mean age at first registration is eight years and nine months, for about 70% of children with special needs the first onset occurs within three years after entering primary school and for more than 90% by the end of primary school.

In terms of severity, we know from the SPS staff that children with more severe special needs have longer contact histories and usually more complex therapy settings. Following this reasoning, we use the number of consultations with the SPS for each child as a measure of the severity of special needs. The average number of consultations with the SPS is 2.6 for the full sample and 8.0 for children who are referred to the SPS.

While we have data on school performance for all children, we have information on students' post-compulsory education choice for approximately 82% of the sample. For labor market outcomes, we have employment and earnings data for 55% of the sample. The higher attrition rates for later outcomes are primarily mechanical, due to many students not yet having finished compulsory school. For these students we observe neither their post-compulsory education choice nor their labor market outcomes. As we discuss and show in section 4.5, attrition rates are unrelated to the treatment (day of birth), balanced around the cutoff, and essentially zero for all but the youngest cohorts.

The average worker earns 1,900 Swiss francs[8] per month and is employed for approximately ten and a half months per year. The earnings we observe are lower than the Swiss average, because we observe individuals at an early stage in their career. The wages reported in the present study are earned when the workers are between 17 and 24. Most of the earnings we observe are therefore apprenticeship wages, not only because 65% of each Swiss cohort follows the vocational track but also because few university graduates enter the labor market before 24.

Regarding DI, we identified 309 DI recipients (1.1%), a proportion that is consistent with aggregate statistics on the prevalence of DI among young adults in Switzerland. While we observe neither the type of disability nor the disability degree, we know the amount of first pillar DI benefits transferred, which is on average 18,136 Swiss Francs per DI recipient per year.

3 Empirical Strategy

The school starting cutoff date causes some children to be older than others when entering compulsory education. We adopt a regression discontinuity (RD) design around the August 1 cutoff to study the effect of school starting age on the development of special needs in early childhood (Hahn, Todd, and Van der Klaauw, 2001). The parameter of interest is the local average treatment effect at the cutoff τ_{RD}. We estimate τ_{RD} nonparametrically by approximating the regression functions above and below the cutoff via local linear regressions, with weights computed by applying a triangular kernel function on the distance of each observation's score to the cutoff. This nonparametric local polynomial approach has become the standard choice for estimation of RD

[8]In 2018, 1 Swiss franc was valued at 1.01 USD.

Table 1: Descriptive Statistics

	(1) Mean	(2) SD	(3) Min	(4) Max	(5) N
(a.1) Special needs: Incidence, referral, onset					
Referred to the SPS	0.323	0.468	0.00	1.00	52,082
Referred before primary school	0.066	0.248	0.00	1.00	52,082
Referred during primary school	0.257	0.437	0.00	1.00	52,082
Dismissed after initial examination	0.044	0.205	0.00	1.00	52,082
SN diagnosis	0.279	0.448	0.00	1.00	52,082
Consultations with the SPS	2.592	5.513	0.00	144	52,082
(a.2) Special needs: Type					
Learning impairment	0.196	0.397	0.00	1.00	52,082
Behavioral problem	0.070	0.255	0.00	1.00	52,082
Speech impediment	0.132	0.338	0.00	1.00	52,082
Dyslexia/dyscalculia	0.105	0.306	0.00	1.00	52,082
ADHD	0.031	0.174	0.00	1.00	52,082
Physical disability	0.006	0.076	0.00	1.00	52,082
(b) School performance					
Test score (grade 8): Composite (standardized)	0.000	1.000	-4.30	4.09	52,082
Test score (grade 8): Math (standardized)	0.000	1.000	-4.27	3.71	52,082
Test score (grade 8): German (standardized)	0.000	1.000	-4.23	3.89	52,082
Bridge year between KG and PS	0.050	0.218	0.00	1.00	52,082
Grade repetition by 8th grade	0.017	0.130	0.00	1.00	52,082
High track in secondary school	0.621	0.485	0.00	1.00	52,082
(c) Later outcomes					
Vocational education	0.643	0.479	0.00	1.00	42,452
Academic preparation track	0.181	0.385	0.00	1.00	42,452
Employment (months per year)	10.62	2.288	1.00	12.0	28,679
Monthly wage	1,871	964.4	300	4,825	28,679
DI recipient	0.011	0.103	0.00	1.00	28,561
(d) Covariates					
Female	0.495	0.500	0.00	1.00	52,082
Non-native speaker	0.155	0.362	0.00	1.00	52,082
Born after July 31	0.410	0.492	0.00	1.00	52,082
Age at SPS registration	8.736	2.190	3.18	14.0	16,829

Notes: Descriptive statistics for the main estimation sample. Data are from the School Psychological Service St. Gallen, the Ministry of Education of the canton of St. Gallen, the Stellwerk test service provider, the Swiss Federal Statistical Office, and the Swiss Central Compensation Office.

treatment effects (Gelman and Imbens, 2018).[9]

For the choice of bandwidth, we follow a recent approach developed in Calonico, Cattaneo, and Titiunik (2014) and Calonico et al. (2018). They show that commonly used bandwidth selectors tend to yield bandwidths that are too large to ensure the validity of the underlying distributional approximations, potentially leading to non-negligible bias. They propose an alternative method, with the RD point estimate corrected by an estimated bias term; the standard error estimates are then adjusted for the additional variability resulting from the estimation of the bias correction term. Throughout the paper, we present the bias-corrected estimates, and we select the bandwidth such that the point estimator for the bias-corrected estimate is mean-square-error optimal (see Calonico et al., 2018). Given that we have the exact birth date for each individual, the bandwidth is measured in days.

Although predetermined individual characteristics are not required for identification, their inclusion may improve precision. Identification is valid if the conditional expectation functions of the covariates are continuous at the cutoff (Calonico et al., 2018). In some regressions, we include gender, an indicator for non-native speaker, birth-year cohort fixed effects[10], postal code, and test year fixed effects. Including covariates does not change the results qualitatively, and in our main specification we only include gender, non-native speaker, and birth-year cohort fixed effects.

Identification in the RD design relies on the idea of local randomization around the threshold. One main concern is that individuals manipulate the running variable by systematically timing birth in consideration of the school starting threshold. Manipulation typically leads to asymmetric sample selection and sorting on either side of the cutoff, which is often indicated by bunching in the distribution on one side of the assignment threshold. Appendix Figure A1 shows the distribution of date of birth in our sample. There is no visible change in births before August 1. More formally, using the test outlined in McCrary (2008), we cannot reject the null hypothesis of a zero discontinuity at the threshold (p-value = 0.819).

In addition, if observations are locally randomized at the threshold, any predetermined characteristics should be balanced at the threshold. Panels (a) and (b) in Appendix Figure A2 show discontinuity graphs for the probability of being female and the probability of being a non-native speaker, respectively. Both characteristics are balanced at the threshold (the p-values of the RD estimate for gender and non-native speaker are 0.820 and 0.898, respectively). To alleviate residual concerns about the covariate balance, the main regression results control for gender, non-native speaker, and birth cohort fixed effects.

We complement the reduced form estimates with a two-sample instrumental variable strategy (TSIV). This approach is a fuzzy RD design, with first-stage and reduced-form calculated from two separate samples. We base our approach on theoretical work by Angrist and Krueger (1992) and Inoue and Solon (2010), along with the applied studies by Dee and Evans (2003), Devereux and Hart (2010), and Van den Berg, Pinger, and Schoch

[9] In implementing the RD approach, we need to choose the kernel function for weighting the observations and the bandwidth for determining the sample size around the cutoff. The choice of the kernel function makes little difference in practice. For our main specifications, we rely on a triangular kernel, which is better suited for estimating a function at boundary points than the Epanechnikov kernel (Cheng, Fan, and Marron, 1997).

[10] Note that the optimal bandwidth never exceeds the five-month window.

(2016).

Because our model is exactly identified, it is straightforward to show that dividing the reduced-form parameter τ_{RD} by the first stage is equivalent to the TSIV estimator. The first stage effect (denoted as γ_{RD}) is calculated by regressing the date of birth on the actual SSA.[11] The complier population of this instrument thus comprises children who enter school at an older age because of their date of birth. The following expression provides an estimate for the effect of SSA on the outcome of interest Y_i:

$$\hat{\beta}_{\text{RD}} = \hat{\tau}_{\text{RD}}/\hat{\gamma}_{\text{RD}} \quad (1)$$

Standard errors for $\hat{\beta}_{\text{RD}}$ are computed using bootstrap, a common approach in the literature on TSIV (Björklund and Jäntti, 1997; Van den Berg, Pinger, and Schoch, 2016). The underlying assumption is that the covariance between the first-stage and reduced-form estimates is zero. This assumption is plausible in our application, because reduced-form and first-stage estimates stem from different cohorts (1992-2003 for the reduced from and 2006-2011 for the first stage).

SN referral and diagnosis is a multi-stage process. In the interpretation of the results, we assume that experts diagnose without error (Dalsgaard et al., 2012). Errors can still occur at the referral stage. If these errors are absent or not affected by SSA, we can interpret our estimates as the effect on the underlying incidence of SN. In contrast, if SSA affects referral errors, additional assumptions are required to identify or bound the effect of SSA on the underlying incidence of (true) SN status. If we rule out false-negative non-referrals, and assume that teachers only commit false positive referral errors (over-referral of non-SN students) under either treatment arm, our analysis using expert diagnoses as an outcome identifies the effect of SSA on the true underlying incidence of SN. Alternatively, changes in the referral propensity may involve a reduction in false negative referral errors, changing interpretation as we identify a mixture of detection effects and changes in underlying SN incidence. However, since there are low costs associated with referral and the harm of referral errors is asymmetric, we believe that false negative referrals are rare. Note that even in the presence of false negative referrals, our estimates can always be interpreted as the causal effect of SSA on SN diagnoses.

4 Results

This section presents the results of the paper and the robustness checks. We defer the graphical analysis to Appendix Figures A3 to A7.

[11]The first stage is estimated following the exact same procedure as for the reduced form, i.e., nonparametrically by approximating the regression functions above and below the cutoff via local linear regressions, with weights computed by applying a triangular kernel function on the distance of each observation's score to the cutoff.

4.1 Special Needs Diagnosis, Onset, and Type

We first present the reduced form estimation results of the effect of school starting age on the development of special needs. The analysis is structured in three parts, presented in Table 2. First, we focus on the probability of being *referred* to the SPS. The decision to refer a child to the SPS is generally made by the teacher and does not necessarily turn into a diagnosis. For this reason, in the second part we examine special needs diagnoses, which are performed by SPS psychologists. This allows us to separate potential over-referring behavior towards younger children and actual special needs diagnoses. Finally, in part three we analyze special needs types in detail, to understand whether a particular health condition is more likely to occur among children entering school at a younger age. Table 2 shows the estimated threshold effects using local linear regressions. We control in all regressions for gender, non-native speaker, and year-of-birth fixed effects. Observations are weighted using a triangular kernel, and the bandwidth is symmetric around the threshold and chosen by minimizing the regression discontinuity mean squared error.

Panel (a) in Table 2 presents the point estimates for the SSA effect on SPS referrals. Being born on August 1 instead of July 31 decreases the probability of being referred for special needs by 5.7 percentage points, corresponding approximately to a 16% reduction. This result could be driven by increases in screening, which may occur if teachers are inclined to refer children for special needs evaluation because of their younger age rather than because of any actual need for special education. To investigate this possibility, column (2) focuses on children who are referred to the SPS but dismissed after initial examination by SPS professionals. We find significantly higher rates of registered cases without a diagnosis among children born before August 1, which suggests that teachers tend to over-refer younger children to the SPS. In terms of timing of the SPS referral, column (3) shows no discontinuity in the incidence of referrals occurring before or during kindergarten. In contrast, as column (4) indicates, the entire discontinuity in SPS registrations consists of referrals occurring during primary school. This is an important result, because it underscores that excess referrals towards younger children coincide with children entering a more competitive educational environment.[12] In terms of reason for registration, the difference at the cutoff is exclusively due to referrals due to behavioral problems and not learning impairments.

Panel (b) presents the effect of SSA on special needs diagnoses. At the cutoff, the probability of being diagnosed with special needs decreases by 4.2 percentage points. This means children born immediately after August 1 are approximately 14% less likely to have a special needs diagnosis than their peers born shortly before August 1. Panel (b) also examines the timing of the diagnosis (columns 3 and 4) and the type of special needs (columns 5 and 6). Regarding the timing of the diagnosis, we find – as for SPS referrals – that younger children are more likely to develop special needs exclusively after primary school entry. Most of the referrals and diagnoses occur between the ages of eight and nine, when pupils start receiving school grades (second semester

[12]This interpretation is based on two elements. First, students transition from an informal, play-based learning environment in kindergarten to formal schooling in primary school. Second, students receive school marks for the first time in grade two, when they are about 8-9 years old. This corresponds to the average age of referral (8.7, Table 1), suggesting that special needs become more salient as relative performance starts to matter and differences among students are more visible.

Table 2: Reduced-Form Effect of SSA on Special Needs

(a) Referral to the SPS

	(1)	(2)	(3)	(4)	(5)	(6)
	Referred to the SPS	No diagnosis, dismissal	Before school	During school	Learning impairment	Behavioral problem
τ_{RD}	-0.057***	-0.018**	0.017	-0.072***	-0.009	-0.031**
	(0.020)	(0.009)	(0.012)	(0.017)	(0.019)	(0.013)
Bandwidth (days)	48	62	42	52	42	43
$\bar{Y}_{-\text{bw}}$	0.341	0.056	0.068	0.274	0.226	0.089
$N_{-\text{bw}}$	6,890	9,027	5,988	7,451	6,160	6,160
$N_{+\text{bw}}$	6,830	9,215	5,936	7,480	6,081	6,081
N	52,082	52,082	52,082	52,082	52,082	52,082

(b) Special needs: Diagnosis

	(1)	(2)	(3)	(4)	(5)	(6)
	SN diagnosis	Nr. of consultations	Diagnosis before school	Diagnosis during school	Learning impairment	Behavioral problem
τ_{RD}	-0.042**	-0.672***	0.022**	-0.064***	-0.011	-0.026**
	(0.020)	(0.255)	(0.009)	(0.018)	(0.019)	(0.012)
Bandwidth (days)	45	43	47	45	38	44
$\bar{Y}_{-\text{bw}}$	0.285	2.737	0.040	0.245	0.197	0.075
$N_{-\text{bw}}$	6,478	6,321	6,890	6,616	5,417	6,321
$N_{+\text{bw}}$	6,401	6,244	6,830	6,541	5,364	6,244
N	52,082	52,082	52,082	52,082	52,082	52,082

(c) Special needs: Type

	(1)	(2)	(3)	(4)	(5)	(6)
	Speech impediment	Speech imped. (excl. dys.)	Dyslexia/ dyscalculia	ADHD	Physical disability	Domestic violence
τ_{RD}	-0.031*	-0.028**	-0.015	-0.009	0.001	0.001
	(0.016)	(0.012)	(0.014)	(0.008)	(0.003)	(0.001)
Bandwidth (days)	37	41	40	44	45	32
$\bar{Y}_{-\text{bw}}$	0.132	0.086	0.102	0.034	0.004	0.003
$N_{-\text{bw}}$	5,280	5,862	5,714	6,478	6,616	4,699
$N_{+\text{bw}}$	5,204	5,797	5,649	6,401	6,541	4,594
N	52,082	52,082	52,082	52,082	52,082	52,082

Notes: Estimates for τ_{RD} correspond to the treatment effect derived in section 3. All models include birth cohort specific effects and indicators for gender and non-native speaker. Heteroskedasticity-robust standard errors are reported in parentheses. *, **, and *** denote significance at the 10%, 5% and 1% level, respectively. Data are from the School Psychological Service St. Gallen, the Ministry of Education of the canton of St. Gallen, the Stellwerk test service provider, the Swiss Federal Statistical Office, and the Swiss Central Compensation Office.

of second grade). Again, the difference at the cutoff is entirely driven by diagnoses of behavioral problems, as we find no effect for learning impairments. Interestingly, the point estimates for behavioral problems are quite similar to those found for Florida by Dhuey et al. (2019), who show that September-born children have a 2.5-3.4% higher probability of being classified as having a "behavioral disability" (see Dhuey et al., 2019, Table 3, page 555).

In terms of special needs severity, which we measure using the number of consultations with the SPS[13], children born just after August 1 require approximately 0.7 consultations less than children born before the cutoff date (panel b, column 2). A smaller number of consultations may result from either less severe special needs or lower incidence of special needs. To understand which of the two explains the results, one might repeat the RD analysis while conditioning on being referred to the SPS. Doing so could shed light on the mechanism but would also put the validity of the RD design at risk, because it potentially induces differential selection on each side of the cutoff. Nonetheless, we provide the estimates as descriptive evidence on the average number of consultations per referred child on either side of the cutoff. Children referred to the SPS born in the last week of July have on average 8.55 consultations, while their peers born in the first week of August have approximately 7.73 consultations. This is nearly a 10% difference and, although it does not have a causal interpretation, it suggests that younger children are not only more likely to be referred and diagnosed but also suffer from more serious special needs.

A discontinuity also exists for diagnoses made before primary school entry (panel b, column 3). This result is puzzling, but it can be explained by inspecting the type of diagnoses on the right-hand-side of the cutoff. More than two-thirds of the cases on the right of the cutoff are requests starting kindergarten ahead of schedule, a request made by the parents who wish that their children go to kindergarten one year earlier than the regular curriculum intends. Although entering kindergarten ahead of schedule is typically not permitted, exceptions are granted upon formal request by the parents to the SPS. After removing these instances on both sides of the cutoff, the estimated discontinuity is zero ($\tau_{\mathrm{RD}} = -0.001$, standard error $= 0.006$).

To further understand which conditions drive the difference around the cutoff, panel (c) examines the incidence of the most common special needs diagnoses, and in addition the incidence of physical disability and domestic violence. We include physical disability and domestic violence in the analysis as a placebo check, because these two conditions should be unrelated to SSA. The first two columns of panel (c) focus on speech impediments. Since overlap between speech impediment and dyslexia/dyscalculia exists, we distinguish between speech impediments including dyslexia/dyscalculia and speech impediments excluding dyslexia/dyscalculia. Entering school at an older age significantly decreases speech impediments by 3.1 percentage points (approximately 20%). The effect is more precisely estimated and larger (in relative terms) for speech impediments other than dyslexia/dyscalculia. These speech impediments are typically communication disorders that relate to the difficulty to speak (e.g., apraxia of speech, language disorder), fluency disorders (e.g., stuttering), or muscle

[13]We use the total number of consultations in the main analysis because the SPS staff advised us to do so. The results are robust to the use of alternative measures of SN severity such as spell duration at the SPS or number of consultations per year registered at the SPS.

weakness (e.g., articulation disorders, dysarthria). Note that while dyslexia and dyscalculia are primarily determined by genetic inheritance (Schumacher et al., 2007), the other communication disorders listed typically are not.

Column (4) examines the incidence of ADHD by birth date. ADHD has attracted most of the interest in the literature, partly because the exact causes of ADHD are not well understood. The medical literature considers ADHD a familial disorder, because the frequency of ADHD is approximately five- to sixfold greater among first-degree relatives than in the general population.[14] Despite the hereditary factor, multiple studies (Elder, 2010; Evans, Morrill, and Parente, 2010; Layton et al., 2018) have linked SSA to ADHD. These studies also suggest that the ADHD diagnosis may not be entirely objective and that relative standards are being applied. Evans, Morrill, and Parente (2010) argue that part of the problem may be that who performs the diagnosis is often a family doctor and not a specialist. In fact, when specialists establish the diagnosis, the prevalence of ADHD is much smaller (around 1.6% for Denmark, according to Dalsgaard et al., 2012). In our data, diagnoses are performed by child psychologists and the ADHD prevalence we observe is 3%, about a third of the U.S. prevalence reported in Elder (2010) and Evans, Morrill, and Parente (2010). In line with Dalsgaard et al. (2012), we find no clear jump in ADHD prevalence at the cutoff. We also find no effect for physical disability (column 5) and domestic violence (column 6), which are outcomes determined outside of school and unrelated to SSA.

Overall, the effect of SSA on behavioral problems is the main driver of the results, because being born on August 1 instead of July 31 increases the probability of developing behavioral problems after school entry by 35%. As the administrative records of the SPS reveal, there are three main categories of behavioral problems: socio-emotional problems, disruptive behavior, and violent behavior. It appears thus that children starting school at a younger age tend to display a lack of maturity in their behavior, as suggested by developmental psychologists (Whitebread, 2012). Our results are also consistent with Dee and Sievertsen (2018), who show that a one-year delay in the start of school reduces inattention and hyperactivity at age seven.

4.2 Education and Labor Market Outcomes

Early differences in the development of special needs can lead to persistent differences in achievement, human capital investments, and labor market outcomes. Understanding whether SSA generates health inequality between SN and non-SN students is a key policy question. On the one hand, more educated individuals have better health later in life (Cutler and Lleras-Muney, 2010). On the other hand, children registered with the SPS typically receive support, and they may also repeat a grade if the gap in achievement is too large for them to bridge. Educational achievement differences between younger and older children may thus fade away over time (Crawford, Dearden, and Greaves, 2014). In this section, we examine whether the differences in SSA have a lasting impact on school performance, post-compulsory education trajectories, employment, earnings, and disability status.

In Table 3, we test whether differences still persist at the age when children are about to complete sec-

[14] See, for example, Biederman et al. (1992), Smalley (1997), and Thapar et al. (1999).

ondary school and take a compulsory standardized test (around age 15). To remove possible grading effects, the regressions additionally control for the year the test was administered. We find that children born on August 1 or after consistently outperform younger students. Their test scores in Math, German, and a composite score of both are on average 0.1 standard deviations better than those of children born on July 31 or before. Although we cannot separate the SSA effect from the age-at-test effect, our results corroborate the findings in the literature (Attar and Cohen-Zada, 2018; Bedard and Dhuey, 2006; Black, Devereux, and Salvanes, 2011; Fredriksson and Öckert, 2014).

Table 3: Reduced-Form Effect of SSA on Educational Outcomes

	(1)	(2)	(3)	(4)	(5)	(6)
	STANDARDIZED TEST IN GRADE 8			GRADE RETENTION AND TRACKING		
	Composite	Math	German	Bridge year btw. KG and PS	Grade repetition by 8th grade	Higher track
τ_{RD}	0.103**	0.072*	0.119***	-0.028***	-0.003	0.014
	(0.042)	(0.043)	(0.041)	(0.008)	(0.005)	(0.022)
Bandwidth (days)	44	45	44	56	68	42
$\bar{Y}_{-\text{bw}}$	0.005	0.006	0.004	0.059	0.018	0.615
$N_{-\text{bw}}$	6,478	6,478	6,321	8,060	9,721	6,160
$N_{+\text{bw}}$	6,401	6,401	6,244	8,116	9,922	6,081
N	52,082	52,082	52,082	52,082	52,082	52,082

Notes: Estimates for τ_{RD} correspond to the treatment effect derived in section 3. All models include birth cohort specific effects and indicators for gender and non-native speaker. Models in columns 1, 2, and 3 also control for year of test. Heteroskedasticity-robust standard errors are reported in parentheses. *, **, and *** denote significance at the 10%, 5% and 1% level, respectively. Data are from the School Psychological Service St. Gallen, the Ministry of Education of the canton of St. Gallen, the Stellwerk test service provider, the Swiss Federal Statistical Office, and the Swiss Central Compensation Office.

We also investigate tracking in secondary school and two distinct grade retention outcomes. The first measure is whether a child was assigned to a bridge year between kindergarten and primary school. The purpose of a bridge year is to smooth the transition from kindergarten to the school environment. The second measure is whether a child repeated a grade by the end of secondary school. While we do not find that younger children have a higher risk of failing a grade, younger children are 47% more likely to do a bridge year between kindergarten and primary school than their older peers. This result indicates that many children starting school at a younger age will eventually prolong their compulsory education by one year. A flexible school system which allows children to do so might be key to alleviate disadvantages of younger children when entering the labor market. Adding this additional year early in the school career might be preferable to later grade repetition, since it avoids early disadvantages which persist through several grades or stigma effects associated with failing a grade.

In terms of tracking, we find no significant discontinuity at the cutoff. This result seems to be at odds with Mühlenweg and Puhani (2010), who find that relatively young German students are only two-thirds as likely as older students to be assigned to the academic track. However, tracking in Switzerland occurs two years later than in Germany (age 12 instead of 10). In addition, the German school system facilitates mobility between tracks only around age 16, whereas in St. Gallen switching tracks is allowed already at age 14. These

two institutional differences combined might explain the difference in results. In fact, Mühlenweg and Puhani (2010) find that the school-entry age effect on track attendance is indeed mitigated at age 16, when educational institutions facilitate track modification.

Table 4 presents the results for post-compulsory education choices. The table focuses on five outcomes defined as follows. First, a summary measure of whether a student entered any post-compulsory education track. Second, whether a student entered the academic track (high school) versus everything else. Third, whether a student entered the VET track versus everything else. Fourth, whether a student selected the low-quality VET track, compared to everything else.[15] Fifth, whether a student entered the high-quality VET track versus everything else. The analysis of post-compulsory education trajectories can also be performed by conditioning on downstream or earlier outcomes (i.e., conditioning on having chosen any post-compulsory education for instance). Appendix Table A1 presents estimates from this alternative approach.

Overall, we do not find that achievement differences persist into differences in success at transition to post-compulsory education. Children entering school early are equally likely to enter VET as children entering school later. Similarly, younger children are also similarly likely to enter the academic preparation track. However, we find that among students entering the vocational track, being born just after the cutoff date increases the probability of choosing a high-quality vocational track. Although SSA does not influence the choice between academic or vocational education, SSA affects the type of VET students choose.

Table 4: Reduced-Form Effect of SSA on Post-Compulsory Education Choice

	(1)	(2)	(3)	(4)	(5)
	Post-compulsory education started	Academic track	Vocational track	Low-quality VET	High-quality VET
τ_{RD}	0.016	-0.008	0.037	-0.042***	0.063**
	(0.020)	(0.016)	(0.029)	(0.016)	(0.031)
Bandwidth (days)	41	32	29	37	26
$\bar{Y}_{-\text{bw}}$	0.826	0.151	0.646	0.095	0.580
$N_{-\text{bw}}$	4,749	3,666	3,429	4,274	3,067
$N_{+\text{bw}}$	4,751	3,645	3,426	4,263	3,065
N	42,452	42,452	42,452	42,452	42,452

Notes: Estimates for τ_{RD} correspond to the treatment effect derived in section 3. All models include birth cohort specific effects and indicators for gender and non-native speaker. Heteroskedasticity-robust standard errors are reported in parentheses. *, **, and *** denote significance at the 10%, 5% and 1% level, respectively. Data are from the School Psychological Service St. Gallen, the Ministry of Education of the canton of St. Gallen, the Stellwerk test service provider, the Swiss Federal Statistical Office, and the Swiss Central Compensation Office.

For individuals who entered the labor market between 2007 and 2016, we have information about their earnings and employment. To make labor market outcomes more comparable, we group these outcomes relative to the end of compulsory schooling. That is, we examine employment and earnings one year after compulsory school, two years after compulsory school, and so on, up to seven years after compulsory school end (the maximum we observe in the data).

[15] Switzerland has two tracks within the vocational system. The higher quality track lasts three to four years and is comprised of jobs requiring extensive skills and competences (for example, computer programmer or nurse). The lower quality track lasts two years and is restricted to jobs requiring basic skills (for example, painter or plumber). For many jobs, both a high- and a low-quality track is available.

Figure 1 summarizes the results, divided into employment (panel a) and earnings (panel b). As before, estimations control for birth year fixed-effects, gender, and nonnative speaker, and are based on triangular kernel weights and a bandwidth choice following Calonico et al. (2018). We find that SSA has no effect on either the number of months per year worked or monthly earnings. The point estimates are all relatively small in size and similar to each other, suggesting the effect is a true zero. These results confirm findings by Dustmann, Puhani, and Schönberg (2017), who find no effects of SSA on experience-adjusted wages and employment in Germany.[16]

The final outcome we consider is early entry into disability insurance. We perform the RDD analysis using DI recipient status as dependent variable and find no effect of SSA on disability status ($\tau_{\mathrm{RD}} = 0.003$, standard error = 0.004).

In sum, our analysis of the effects of SSA indicates that students starting school at a younger age are more likely to develop behavioral problems after primary school entry. This effect diminishes over time and, although present for test scores in eighth grade, disappears after compulsory schooling and does not influence labor market outcomes or disability insurance entry.[17]

4.3 Two-Sample Instrumental Variable Estimation

The previous results are reduced-form estimates, because in our main data set we do not observe actual school starting age. This is a common problem in the SSA literature (e.g. Dalsgaard et al., 2012) and Evans, Morrill, and Parente (2010). In this section, we follow a two-sample instrumental variable approach (TSIV) and instrument actual school starting age with the cutoff rule.

We estimate a first stage based on official education statistics (SDL, *Statistik der Lernenden*). The SDL collects administrative records of the universe of Swiss students since 2010. The data comprise the population of children who were born between 2006 and 2011 and started compulsory education between 2010 and 2016. The raw data for St. Gallen contain 29,580 children, but we exclude those in special schools (820 children) and those with implausible age at kindergarten start (62 children). The final data set contains 28,698 observations, approximately 4,100 children entering compulsory education each year.

Note that the cohorts covered by the SDL (2006-2011) are not the same cohorts covered by our main data set (1992–2003). Although relying on two different cohort samples makes the assumption of zero covariance between the first-stage and reduced-form estimates more plausible, we have to assume that the first stage effect from the SDL generalizes to older cohorts. To determine how credible this latter assumption is, we proceed as

[16] Dustmann, Puhani, and Schönberg (2017) use the school cutoff to instrument for track attendance during compulsory schooling. Performing their reduced form analysis in our data with a binary outcome for being in the high track in secondary school yields an insignificant effect of 0.014 (p-value = 0.532). The exclusion restriction in the study by Dustmann, Puhani, and Schönberg (2017) states that SSA influences wages and employment exclusively through tracking. Our main findings on special needs outcomes challenge the validity of this exclusion restriction (at least for Switzerland), because we report strong effects on the development of special needs arising before tracking occurs.

[17] In terms of heterogeneity, we checked for gender differences across outcomes and distributional effects for test scores. RDD quantile treatments effects are computed following Frandsen, Frölich, and Melly (2012). We generally find no clear heterogeneity pattern: SSA effects are similar for both genders and the quantile treatment effects are constant over the test score distribution. These additional results are available on request from the authors.

Figure 1: The Effect of SSA on Labor Market Outcomes

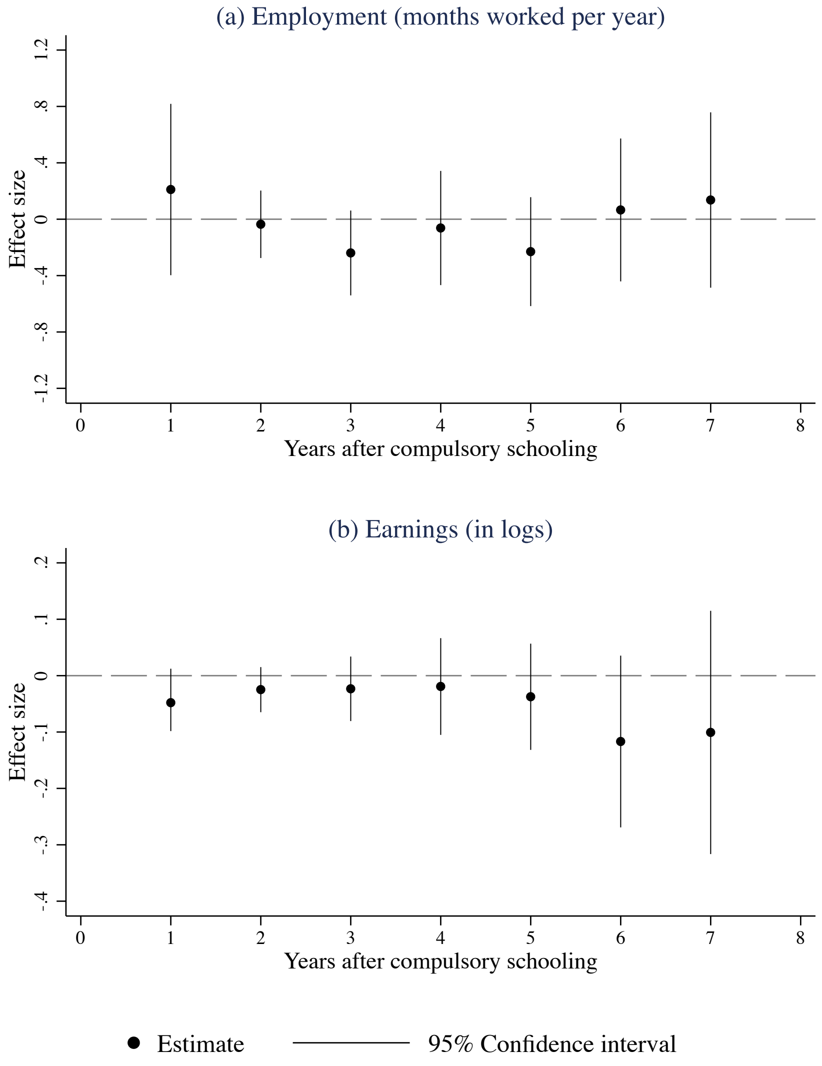

Notes: Local linear regression results are based on triangular kernel weights and a bandwidth choice following Calonico et al. (2018). Earnings are on a logarithmic scale. Sample sizes are as follows: 5,651 for the first year, 19,220 for the second year, 21,591 for the third year, 17,860 for the fourth year, 13,699 for the fifth year, 9,161 for the sixth year, and 4,488 for the seventh year. Data are from the School Psychological Service, the Ministry of Education of the canton of St. Gallen, the Stellwerk test service provider, the Swiss Federal Statistical Office, and the Swiss Central Compensation Office.

Figure 2: Graphical First Stage

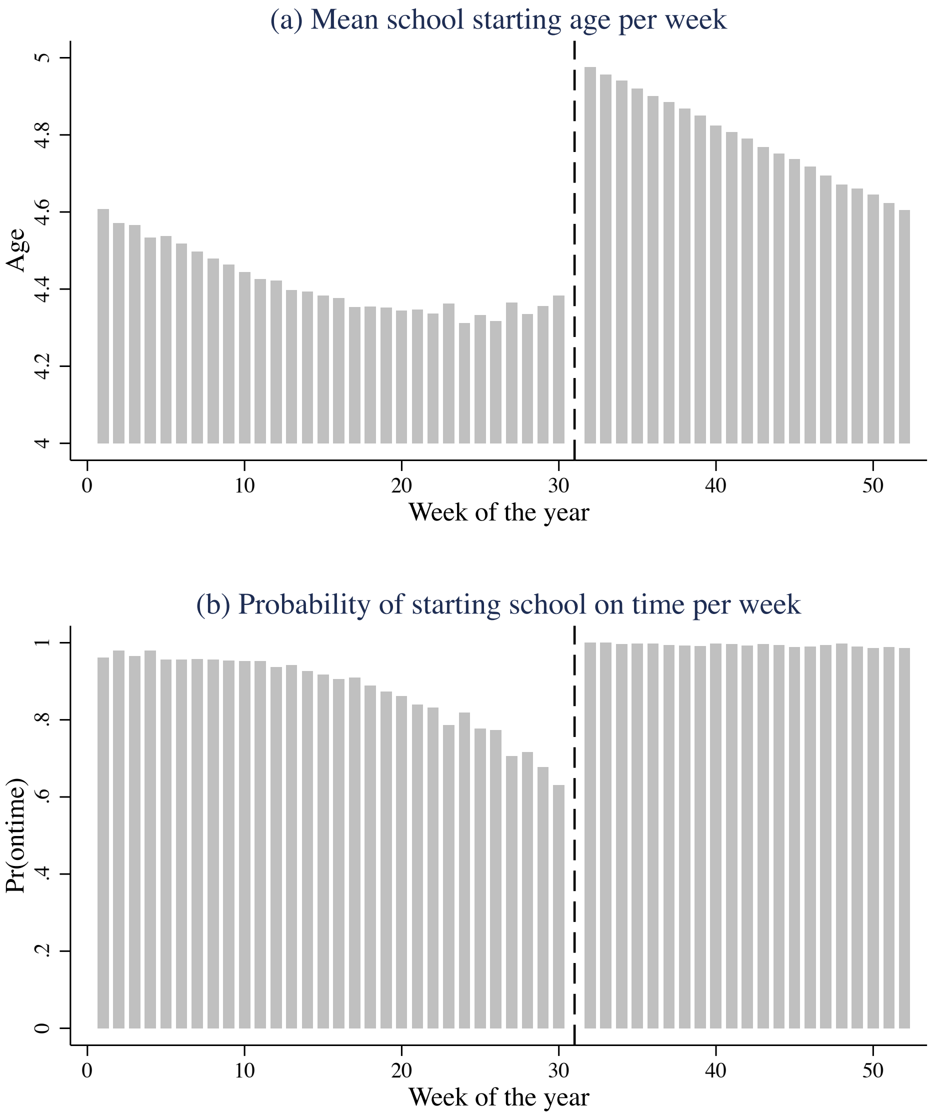

Notes: Each bin represents a calendar week. The vertical dashed line indicates the week of the cutoff. Data are from the Swiss Federal Statistical Office.

follows. First, we include cohort fixed-effects in the calculation of the first stage. This allows us to estimate a within-cohort first stage effect. Second, we estimate one first stage for each cohort and examine whether the first-stage effect is similar across different cohorts. Appendix Figure A8 shows that the first-stage effect is very stable across cohorts, which mitigates the concern of using two different cohorts for reduced form and first stage.

Figure 2 shows the average school starting age for each week of the year (panel a), along with the weekly average probability of starting school on time (panel b). Figure 2 has three findings. First, we observe a continuum of SSAs depending on children's date of birth, from children going to kindergarten when they are barely four years old to children who are already five as they start kindergarten. Second, we observe a clear discontinuity in SSA at the cutoff date. Children born in the week after the cutoff are approximately

seven months older than children born in the week before the cutoff. Third, panel (b) provides some insights on redshirting practices in Switzerland. Redshirting is virtually non-existent for children born either after the cutoff or in the first three months of the year. From April on, the probability of starting school on time decreases from 90% to 63% in the week before the cutoff. The presence of redshirting underscores the importance of dividing the reduced-form estimates by the first stage, in order to estimate a local average treatment effect of SSA.

Appendix Table A2 presents the first-stage estimates using the SDL data. Confirming the findings in Figure 2, the first-stage effect is approximately 0.63***, regardless of the included covariates, the kernel function, the inference method or the bandwidth. The first-stage effect is significant at the 1% level and corresponds to 7.5 months. In other words, children born shortly after the cutoff date start compulsory schooling seven months older than their peers born shortly before the cutoff. Comparing children born in June and July and entering school on time with those born in the same months but entering school late shows redshirting is significantly more common for boys (44%) and native speakers (43%) than for girls (28%) and non-native speakers (18%).

Appendix Table A3 shows the second-stage estimates for special needs (referral, incidence, onset, severity, and type), educational outcomes (test scores, grade retention, and tracking), and post-compulsory education trajectories. We do not present second stage results for labor market outcomes because the reduced-form effect for these outcomes is not statistically significant. Overall, the second stage analysis reinforces the conclusions from the reduced-form estimates. As expected, the second stage coefficients are larger in size and less precisely estimated. For example, being one year older at school start – and thus among the oldest instead of the youngest in class – reduces the probability of being referred to the SPS by 9.1 percentage points (standard error of 0.035) and the probability of developing special needs by 6.7 percentage points (standard error of 0.035). Both effects are relatively large and economically relevant, stemming from special needs developed after school entry and due to behavioral problems and speech impediments. Note that since second stage coefficients are less precisely estimated than those in the reduced form, some effects in Appendix Table A3 do not reach the conventional levels of significance any more. This is the case for over-referring (panel a) and Math test scores (panel d), with p-values of 0.112 and 0.125, respectively.

4.4 Robustness and Validity Checks

We perform a series of robustness checks to demonstrate that our results are stable and not driven by a spurious correlation in the data. First, we perform specification checks using the two main outcomes, namely SPS referral (Appendix Table A4) and special needs diagnosis (Appendix Table A5). In both tables, we perform the analysis without covariates (column 1), then add birth cohort fixed effects (2), individual covariates (column 3, our main specification) and zip code fixed-effects (4). We change the inference to clustering at the running variable (column 5), use an asymmetric optimal bandwidth selection (6), and change the kernel used for weighting (7). None of these modifications change the size or significance of our main estimates.

Second, we perform a set of placebo tests. To do so, we assume a placebo cutoff in the middle of the

distribution of the running variable both left (Appendix Table A6) and right (Appendix Table A7) of the original cutoff. We repeat the analysis for all outcomes used in Table 2. One estimate out of 36 is significant at 5%, that is, no more than what we should expect by chance. Third, we verify that our results are not driven by a specific bandwidth choice. Although such concerns are mitigated by our algorithm-based bandwidth selections, we test the stability of our estimates and the bias-variance trade-off inherent to bandwidth choice by repeating our analysis for a large set of outcomes and bandwidths. Results are plotted in Appendix Figure A9. The graphs indicate no particular pattern deviating from the main results.

Third, we also perform a set of "donut" RD regressions as a third robustness check (Almond and Doyle, 2011; Barreca et al., 2011) to rule out systematic sorting at the cutoff or manipulation of the running variable. The intuition is to omit from the analysis the observations closest to the threshold, where sorting or strategic birth timing is potentially present. We utilize a conservative "donut hole" of 15 days, i.e., one week before the cutoff and one week after. The results are presented in Appendix Table A8. Even though we lose more than two thousands observations closest to the cutoff, the findings are consistent with the main analysis. As expected, the point estimates are less precise due to the loss of observations, but the donut regressions support the validity of the RD design.

Fourth, we conduct a comprehensive attrition analysis. Of the 52,082 individuals, we lose 9,630 (18%) in the post-compulsory education data and 23,403 (45%) in the labor market data. Missing post-compulsory data arises mostly mechanically because some cohorts are too young. This type of attrition is not problematic for the validity of the results. However, if missing information were systematically related to the running variable, this would be a threat to validity. In Appendix Figure A10, panel (a), we plot the attrition rate by cohort, which shows that for the data on education trajectories attrition increases for the last four cohorts and reaches 100% for the two youngest cohorts. This arises mechanically because students who take the standardized test in 2016 would enter post-compulsory education in 2018, but our data on educational trajectories ends in 2016. A similar pattern is present in the labor market data: the curve mechanically increases earlier since most students complete some form of post-compulsory education. Attrition in the labor market data is 100% for the cohorts from 1999 onward, because individuals have not yet started working. Panel (b) of Appendix Figure A10 examines attrition along the running variable, date of birth. Both attrition rates are steady across birth dates. To further investigate the presence of a potential jump, we estimate the discontinuity in attrition rates at the cutoff using the same method as for the main analysis. The results reveal no significant jump at the threshold, with a point estimate (standard error) of 0.004 (0.006) for the education data and 0.005 (0.011) for the labor market data. Overall, we conclude that attrition does not affect the internal validity of the results.

5 Conclusions

The results of the present study suggest that starting school at a relatively younger age is an important factor driving the onset of special needs during the early years of primary school. At the extensive margin, younger

children are more likely to be diagnosed with behavioral problems. At the intensive margin, they receive more frequent examinations and counseling by the school psychologists. Although younger children are more likely to be assigned to a bridge year after kindergarten and children with special needs receive therapies and support, they still score lower than their older peers in standardized tests at the end of compulsory schooling. However, the age differences at school start do not translate into differences in post-compulsory education choices and labor market outcomes. Both younger and older school starters are equally likely to start vocational education or academic preparation, and no significant difference is found for earnings, employment, or entry into disability insurance. Thus, educational achievement differences do not appear to jeopardize the transition after compulsory schooling.

Given the consequences of SSA for early mental health, one important question is whether it matters who determines the SN status of a child. To answer this question, we examine the differences between teachers' referrals to SN services and specialists' diagnoses and find that not every referral is confirmed by a diagnosis. Importantly, we detect a significant difference in over-referring around the cutoff date: children who were born immediately before the cutoff have a 30% higher probability of being dismissed after initial examination. This difference helps explaining the discrepancy in earlier findings for ADHD, where SSA effects are present when ADHD diagnoses are based on non-specialist reports (Evans, Morrill, and Parente, 2010), but disappear when specialists perform the diagnosis (Dalsgaard et al., 2012). Our evidence suggests that relative standards do play a role, but younger children are nonetheless more likely to develop SN upon school entry.

From a policy perspective, simple measures may mitigate the vulnerability of relatively young children who are born just before the cutoff date. Institutional context matters, and it appears that flexibility of the school system is key. Postponing school entry by one year or granting an extra year to bridge between kindergarten and primary school avoids carrying early developmental delays over to compulsory schooling. Although the practice of redshirting already occurs before kindergarten, it is currently initiated exclusively by the parents. Given that parents of high socio-economic status are more likely to redshirt (Bassok and Reardon, 2013), this practice creates disadvantages for younger children from lower socioeconomic background families. Instead, redshirting should be subject to an institutionalized process and external evaluation. For example, to efficiently identify children at risk, one possibility would be improving information sharing between preschool and kindergarten educators. Currently, such information sharing practice is not institutionalized. The bridge year in our setting is a good example for such an institutionalized process at a later stage, because all children are screened for school readiness by kindergarten teachers before being sent to primary school.

As we cannot differentiate between relative and absolute school starting age, investigating whether a general increase in school starting age would lead to a reduction in special needs conditions is outside the scope of this paper. Resolving this issue – possibly by means of a reform of school starting age providing independent variation – would be a valuable complement to our results and should thus be the focus of future research.

References

Almond, Douglas and Joseph Doyle. 2011. "After midnight: a regression discontinuity design in length of postpartum hospital stays." *American Economic Journal: Economic Policy* 3 (3):1–34.

Angrist, Joshua and Alan Krueger. 1992. "The effect of age at school entry on educational attainment: an application of instrumental variables with moments from two samples." *Journal of the American Statistical Association* 87 (418):328–336.

Attar, Itay and Danny Cohen-Zada. 2018. "The effect of school entrance age on educational outcomes: evidence using multiple cutoff dates and exact date of birth." *Journal of Economic Behavior & Organization* 153:38–57.

Barreca, Alan, Melanie Guldi, Jason Lindo, and Glen Waddell. 2011. "Saving babies? Revisiting the effect of very low birth weight classification." *The Quarterly Journal of Economics* 126 (4):2117–2123.

Bassok, Daphna and Sean Reardon. 2013. "Academic redshirting in kindergarten: Prevalence, patterns, and implications." *Educational Evaluation and Policy Analysis* 35 (3):283–297.

Bedard, Kelly and Elizabeth Dhuey. 2006. "The persistance of early childhood maturity: International evidence of long-run age effects." *The Quarterly Journal of Economics* 121 (4):1437–1472.

Biederman, Joseph, Stephen Faraone, Kate Keenan, Jonathan Benjamin, Belinda Krifcher, Cindy Moore, Susan Sprich-Buckminster, Karen Ugaglia, Michael Jellinek, Ronald Steingard, Thomas Spencer, Dennis Norman, Roselyn Kolodny, Ilana Kraus, James Perrin, Martin Keller, and Ming Tsuang. 1992. "Further evidence for family-genetic risk factors in attention deficit hyperactivity disorder: Patterns of comorbidity in probands and relatives in psychiatrically and pediatrically referred samples." *Archives of General Psychiatry* 49 (9):728–738.

Björklund, Anders and Markus Jäntti. 1997. "Intergenerational income mobility in Sweden compared to the United States." *American Economic Review* 87 (5):1009–1018.

Black, Sandra, Paul Devereux, and Kjell Salvanes. 2011. "Too young to leave the nest? The effects of school starting age." *The Review of Economics and Statistics* 93 (2):455–467.

Calonico, Sebastian, Matias Cattaneo, Max Farrell, and Rocio Titiunik. 2018. "Regression discontinuity designs using covariates." *The Review of Economics and Statistics* .

Calonico, Sebastian, Matias Cattaneo, and Rocio Titiunik. 2014. "Robust nonparametric confidence intervals for regression-discontinuity designs." *Econometrica* 82 (6):2295–2326.

Cheng, Ming-Yen, Jianqing Fan, and James Marron. 1997. "On automatic boundary corrections." *The Annals of Statistics* 25 (4):1691–1708.

Conti, Gabriella, James Heckman, and Sergio Urzua. 2010. "The education-health gradient." *American Economic Review P&P* 100 (2):234–38.

Crawford, Claire, Lorraine Dearden, and Ellen Greaves. 2014. "The drivers of month-of-birth differences in children's cognitive and non-cognitive skills." *Journal of the Royal Statistical Society: Series A* 177 (4):829–860.

Cutler, David and Adriana Lleras-Muney. 2010. "Understanding differences in health behaviors by education." *Journal of Health Economics* 29 (1):1–28.

Dalsgaard, Soren, Maria Knoth Humlum, Helena Skyt Nielsen, and Marianne Simonsen. 2012. "Relative standards in ADHD diagnoses: The role of specialist behavior." *Economic Letters* 117 (3):663–665.

Datar, Ashlesha and Michael Gottfried. 2015. "School entry age and children's social-behavioral skills: Evidence from a national longitudinal study of U.S. kindergartners." *Educational Evaluation and Policy Analysis* 37 (3):333–353.

Dee, Thomas and William Evans. 2003. “Teen drinking and educational attainment: evidence from two-sample instrumental variables estimates.” *Journal of Labor Economics* 21 (1):178–209.

Dee, Thomas and Hans Henrik Sievertsen. 2018. “The gift of time? School starting age and mental health.” *Health Economics* 27 (5):781–802.

Devereux, Paul and Robert Hart. 2010. “Forced to be rich? Returns to compulsory schooling in Britain.” *The Economic Journal* 120 (549):1345–1364.

Dhuey, Elizabeth, David Figlio, Krzysztof Karbownik, and Jeffrey Roth. 2019. “School starting age and cognitive development.” *Journal of Policy Analysis and Management* 38 (3):538–578.

Dhuey, Elizabeth and Stephen Lipscomb. 2010. “Disabled or young? Relative age and special education diagnoses in schools.” *Economics of Education Review* 29 (5):857–872.

Duncombe, William and John Yinger. 2005. “How much more does a disadvantaged student cost?” *Economics of Education Review* 24 (5):513–532.

Dustmann, Christian, Patrick Puhani, and Uta Schönberg. 2017. “The long-term effects of early track choice.” *The Economic Journal* 127 (603):1348–1380.

Elder, Todd. 2010. “The importance of relative standards in ADHD diagnoses: Evidence based on exact birth dates.” *Journal of Health Economics* 29 (5):641–656.

Elder, Todd and Darren Lubotsky. 2009. “Kindergarten entrance age and children’s achievement impacts of state policies, family background, and peers.” *Journal of Human Resources* 44 (3):641–683.

Evans, William, Melinda Morrill, and Stephen Parente. 2010. “Measuring inappropriate medical diagnosis and treatment in survey data: The case of ADHD among school-age children.” *Journal of Health Economics* 29 (5):657–673.

Frandsen, Brigham, Markus Frölich, and Blaise Melly. 2012. “Quantile treatment effects in the regression discontinuity design.” *Journal of Econometrics* 168 (2):382–395.

Fredriksson, Peter and Björn Öckert. 2014. “Life-cycle effects of age at school start.” *The Economic Journal* 124 (579):977–1004.

Gelman, Andrew and Guido Imbens. 2018. “Why high-order polynomials should not be used in regression discontinuity designs.” *Journal of Business & Economic Statistics* .

Grossman, Michael. 1972. “On the concept of health capital and the demand for health.” *Journal of Political economy* 80 (2):223–255.

Hahn, Jinyong, Petra Todd, and Wilbert Van der Klaauw. 2001. “Identification and estimation of treatment effects with a regression-discontinuity design.” *Econometrica* 69 (1):201–209.

Hanushek, Eric, John Kain, and Steven Rivkin. 2002. “Inferring program effects for special populations: Does special education raise achievement for students with disabilities?” *The Review of Economics and Statistics* 84 (4):584–599.

Inoue, Atsushi and Gary Solon. 2010. “Two-sample instrumental variables estimators.” *The Review of Economics and Statistics* 92 (3):557–561.

Layton, Timothy, Michael Barnett, Tanner Hicks, and Anupam Jena. 2018. “Attention deficit-hyperactivity disorder and month of school enrollment.” *New England Journal of Medicine* 379 (22):2122–2130.

McCrary, Justin. 2008. “Manipulation of the running variable in the regression discontinuity design: A density test.” *Journal of Econometrics* 142 (2):698–714.

Morrison, Frederick, Denise Alberts, and Elizabeth Griffith. 1997. “Nature-nurture in the classroom: Entrance age, school readiness, and learning in children.” *Developmental Psychology* 33 (2):254–262.

Mühlenweg, Andrea, Dorothea Blomeyer, Holger Stichnoth, and Manfred Laucht. 2012. "Effects of age at school entry (ASE) on the development of non-cognitive skills: Evidence from psychometric data." *Economics of Education Review* 31 (3):68–76.

Mühlenweg, Andrea and Patrick Puhani. 2010. "The evolution of the school-entry age effect in a school tracking system." *Journal of Human Resources* 45 (2):407–438.

OECD. 2008. *Students with Disabilities, Learning Difficulties and Disadvantages – Policies, Statistics and Indicators*. Paris: OECD Publishing.

Oswald, Yvonne and Uschi Backes-Gellner. 2014. "Learning for a bonus: How financial incentives interact with preferences." *Journal of Public Economics* 118:52–61.

Schumacher, Johannes, Per Hoffmann, Christine Schmäl, Gerd Schulte-Körne, and Markus Nöthen. 2007. "Genetics of dyslexia: the evolving landscape." *Journal of Medical Genetics* 44 (5):289–297.

Schwandt, Hannes and Amelie Wuppermann. 2016. "The youngest get the pill: ADHD misdiagnosis in Germany, its regional correlates and international comparison." *Labour Economics* 43:72–86.

Smalley, Susan. 1997. "Genetic influences in childhood-onset psychiatric disorders: autism and attention-deficit/hyperactivity disorder." *The American Journal of Human Genetics* 60 (6):1276–1282.

Stipek, Deborah and Rosaleen Ryan. 1997. "Economically disadvantaged preschoolers: Ready to learn but further to go." *Developmental Psychology* 33 (4):711–723.

Thapar, Anita, Jane Holmes, Kay Poulton, and Richard Harrington. 1999. "Genetic basis of attention deficit and hyperactivity." *The British Journal of Psychiatry* 174 (2):105–111.

Van den Berg, Gerard, Pia Pinger, and Johannes Schoch. 2016. "Instrumental variable estimation of the causal effect of hunger early in life on health later in life." *The Economic Journal* 126 (591):465–506.

Wagner, Mary and Jose Blackorby. 1996. "Transition from high school to work or college: How special education students fare." *The Future of Children* 6 (1):103–120.

Whitebread, David. 2012. *Developmental Psychology and Early Childhood Education: A Guide for Students and Practitioners*. Sage Publications, London (U.K.).

Wolter, Stefan C. and Paul Ryan. 2011. "Apprenticeship." In *Handbook of the Economics of Education*, vol. 3, edited by Erik Hanushek, Stephen Machin, and Ludger Woessmann, chap. 11. Elsevier, 521–576.

Appendix: Supplementary Material

Table A1: Alternative Specification for the SSA Effect on Post-Compulsory Education

	(1)	(2)	(3)
	Post-compulsory education started	Vocational track (vs. academic)	High-quality vocational track
τ_{RD}	0.016	0.030	0.064***
	(0.020)	(0.026)	(0.024)
Bandwidth (days)	41	30	32
$\bar{Y}_{-\text{bw}}$	0.826	0.783	0.860
$N_{-\text{bw}}$	4,749	2,936	2,475
$N_{+\text{bw}}$	4,751	2,909	2,389
N	42,452	34,954	28,343

Notes: Estimates for τ_{RD} correspond to the treatment effect derived in section 3. All models include birth cohort specific effects and indicators for gender and non-native speaker. Heteroskedasticity-robust standard errors are reported in parentheses. *, **, and *** denote significance at the 10%, 5% and 1% level, respectively. Data are from the School Psychological Service St. Gallen, the Ministry of Education of the canton of St. Gallen, the Stellwerk test service provider, the Swiss Federal Statistical Office, and the Swiss Central Compensation Office.

Table A2: First Stage Analysis

	(1)	(2)	(3)	(4)	(5)	(6)
γ_{RD}	0.622***	0.623***	0.627***	0.626***	0.626***	0.627***
	(0.016)	(0.016)	(0.015)	(0.016)	(0.016)	(0.017)
Cohort FE		✓	✓	✓	✓	✓
Indiv. covariates			✓	✓	✓	✓
Kernel	Triangular	Triangular	Triangular	Triangular	Epanechnikov	Triangular
Inference method	Robust	Robust	Robust	Robust	Robust	Cluster
BW selection	Symmetric	Symmetric	Symmetric	Asymmetric	Symmetric	Symmetric
bw_- (days)	63	63	69	66	64	68
bw_+ (days)	63	63	69	17	64	68
$\bar{Y}_{-\text{bw}_-}$	4.358	4.358	4.358	4.358	4.358	4.358
$N_{-\text{bw}_-}$	5,301	5,301	5,787	5,507	5,370	5,587
$N_{+\text{bw}_+}$	5,052	5,052	5,552	1,395	5,113	5,385
N	28,698	28,698	28,698	28,698	28,698	28,698

Notes: Estimates for γ_{RD} are derived by local linear regression with bandwidth choice following Calonico et al. (2018). *, **, and *** denote significance at the 10%, 5% and 1% level, respectively. Data are from the Swiss Federal Statistical Office.

Table A3: Second Stage Effects of SSA

	(1)	(2)	(3)	(4)	(5)	(6)
			(a) REFERRAL TO THE SPS			
	Referred to the SPS	Before school	During school	Learning impairment	Behavioral problem	No diagnosis, dismissal
$\hat{\beta}_{RD}$	-0.091**	0.028	-0.115***	-0.015	-0.049**	-0.028
	(0.035)	(0.020)	(0.033)	(0.032)	(0.023)	(0.018)
			(b) SPECIAL NEEDS: DIAGNOSIS			
	SN diagnosis	Diagnosis before school	Diagnosis during school	Learning impairment	Behavioral problem	Nr. of consultations
$\hat{\beta}_{RD}$	-0.067*	0.035**	-0.101***	-0.017	-0.042**	-1.072**
	(0.035)	(0.016)	(0.031)	(0.031)	(0.021)	(0.442)
			(c) SPECIAL NEEDS: TYPE			
	Speech impediment	Speech imped. (excl. dys.)	Dyslexia/ dyscalculia	ADHD	Physical disability	Domestic violence
$\hat{\beta}_{RD}$	-0.050*	-0.044**	-0.024	-0.014	0.001	0.001
	(0.028)	(0.022)	(0.022)	(0.012)	(0.004)	(0.002)
			(d) EDUCATIONAL OUTCOMES: ACHIEVEMENT, GRADE RETENTION, AND TRACKING			
	Composite test score	Math test score	German test score	Bridge year year	Grade repetition	Higher track
$\hat{\beta}_{RD}$	0.164**	0.114	0.190***	-0.061***	-0.011	0.056
	(0.066)	(0.075)	(0.065)	(0.014)	(0.009)	(0.040)
			(e) POST-COMPULSORY EDUCATION: EDUCATIONAL TRAJECTORIES			
	Post-compulsory education started	Academic track	Vocational track	Low-quality VET	High-quality VET	
$\hat{\beta}_{RD}$	0.025	-0.012	0.060	-0.068**	0.100**	
	(0.033)	(0.033)	(0.041)	(0.028)	(0.041)	

Notes: Estimates for $\hat{\beta}_{RD}$ correspond to the treatment effect derived in equation 1. All models include birth cohort specific effects and indicators for gender and non-native speaker. Standard errors are computed using bootstrap (500 repetitions) and reported in parentheses. *, **, and *** denote significance at the 10%, 5% and 1% level, respectively. Data are from the School Psychological Service St. Gallen, the Ministry of Education of the canton of St. Gallen, the Stellwerk test service provider, the Swiss Federal Statistical Office, and the Swiss Central Compensation Office.

Table A4: Specification Checks for SPS Referral

	(1)	(2)	(3)	(4)	(5)	(6)	(7)
τ_{RD}	-0.056***	-0.059***	-0.057***	-0.057***	-0.057**	-0.057***	-0.058***
	(0.019)	(0.019)	(0.020)	(0.020)	(0.024)	(0.018)	(0.020)
Cohort FE		✓	✓	✓	✓	✓	✓
Indiv. covariates			✓	✓	✓	✓	✓
Postcode FE				✓			
Kernel	Triangular	Triangular	Triangular	Triangular	Triangular	Triangular	Epanechnikov
Inference method	Robust	Robust	Robust	Robust	Cluster	Robust	Robust
BW selection	Symmetric	Symmetric	Symmetric	Symmetric	Symmetric	Asymmetric	Symmetric
bw_{-} (days)	53	52	48	48	48	85	48
bw_{+} (days)	53	52	48	48	48	43	48
$\bar{Y}_{-\text{bw}_{-}}$	0.341	0.341	0.341	0.341	0.341	0.341	0.341
$N_{-\text{bw}_{-}}$	7,597	7,597	6,890	6,890	6,890	12,131	6,890
$N_{+\text{bw}_{+}}$	7,635	7,635	6,830	6,830	6,830	6,244	6,830
N	52,082	52,082	52,082	52,082	52,082	52,082	52,082

Notes: Estimates for τ_{RD} correspond to the treatment effect derived in section 3. *, **, and *** denote significance at the 10%, 5% and 1% level, respectively. Data are from the School Psychological Service St. Gallen, the Ministry of Education of the canton of St. Gallen, the Stellwerk test service provider, the Swiss Federal Statistical Office, and the Swiss Central Compensation Office.

Table A5: Specification Checks for SN Diagnosis

	(1)	(2)	(3)	(4)	(5)	(6)	(7)
τ_{RD}	-0.039**	-0.042**	-0.042**	-0.041**	-0.042*	-0.042**	-0.040**
	(0.020)	(0.020)	(0.020)	(0.020)	(0.023)	(0.018)	(0.019)
Cohort FE		✓	✓	✓	✓	✓	✓
Indiv. covariates			✓	✓	✓	✓	✓
Postcode FE				✓			
Kernel	Triangular	Triangular	Triangular	Triangular	Triangular	Triangular	Epanechnikov
Inference method	Robust	Robust	Robust	Robust	Cluster	Robust	Robust
BW selection	Symmetric	Symmetric	Symmetric	Symmetric	Symmetric	Asymmetric	Symmetric
bw_{-} (days)	48	47	45	45	44	86	45
bw_{+} (days)	48	47	45	45	44	38	45
$\bar{Y}_{-\text{bw}_{-}}$	0.285	0.285	0.285	0.285	0.285	0.285	0.285
$N_{-\text{bw}_{-}}$	7,033	6,754	6,478	6,478	6,321	12,281	6,478
$N_{+\text{bw}_{+}}$	6,990	6,696	6,401	6,401	6,244	5,491	6,401
N	52,082	52,082	52,082	52,082	52,082	52,082	52,082

Notes: Estimates for τ_{RD} correspond to the treatment effect derived in section 3. *, **, and *** denote significance at the 10%, 5% and 1% level, respectively. Data are from the School Psychological Service St. Gallen, the Ministry of Education of the canton of St. Gallen, the Stellwerk test service provider, the Swiss Federal Statistical Office, and the Swiss Central Compensation Office.

Table A6: Placebo Cutoff: Days before August 1, Artificial Cutoff Date on April 18

	(1)	(2)	(3)	(4)	(5)	(6)
	(a) REFERRAL TO THE SPS					
	Referred to the SPS	Before school	During school	Learning impairment	Behavioral problem	Nr. of consultations
τ_{RD}	0.032	0.003	0.028	0.034	0.009	0.122
	(0.027)	(0.014)	(0.024)	(0.026)	(0.015)	(0.266)
Bandwidth (days)	29	34	31	26	31	44
$N_{-\text{bw}}$	4,062	4,954	4,363	3,628	4,363	6,417
$N_{+\text{bw}}$	4,405	5,243	4,687	3,946	4,687	6,617
N	30,571	30,571	30,571	30,571	30,571	30,571
	(b) SPECIAL NEEDS: DIAGNOSIS					
	SN diagnosis	Diagnosis before school	Diagnosis during school	Learning impairment	Behavioral problem	No diagnosis, dismissal
τ_{RD}	0.023	0.003	0.021	0.022	0.010	0.010
	(0.026)	(0.012)	(0.024)	(0.025)	(0.014)	(0.010)
Bandwidth (days)	29	36	29	25	31	41
$N_{-\text{bw}}$	4,062	5,124	4,062	3,485	4,363	5,839
$N_{+\text{bw}}$	4,405	5,378	4,405	3,814	4,687	6,055
N	30,571	30,571	30,571	30,571	30,571	30,571
	(c) SPECIAL NEEDS: TYPE					
	Speech impediment	Speech imped. (excl. dys.)	Dyslexia/ dyscalculia	ADHD	Physical disability	Domestic violence
τ_{RD}	-0.009	-0.005	0.003	0.001	0.003	0.001
	(0.017)	(0.015)	(0.015)	(0.009)	(0.003)	(0.003)
Bandwidth (days)	37	33	36	25	32	24
$N_{-\text{bw}}$	5,273	4,639	5,273	3,485	4,498	3,485
$N_{+\text{bw}}$	5,513	4,984	5,513	3,814	4,839	3,814
N	30,571	30,571	30,571	30,571	30,571	30,571

Notes: Estimates for τ_{RD} correspond to the treatment effect derived in section 3. All models include birth cohort specific effects and indicators for gender and non-native speaker. Heteroskedasticity-robust standard errors are reported in parentheses. *, **, and *** denote significance at the 10%, 5% and 1% level, respectively. Data are from the School Psychological Service St. Gallen, the Ministry of Education of the canton of St. Gallen, the Stellwerk test service provider, the Swiss Federal Statistical Office, and the Swiss Central Compensation Office.

Table A7: Placebo Cutoff: Days after August 1, Artificial Cutoff Date on October 15

	(1)	(2)	(3)	(4)	(5)	(6)
	(a) REFERRAL TO THE SPS					
	Referred to the SPS	Before school	During school	Learning impairment	Behavioral problem	Nr. of consultations
τ_{RD}	-0.005	0.006	-0.007	0.011	0.010	-0.163
	(0.030)	(0.017)	(0.025)	(0.026)	(0.018)	(0.304)
Bandwidth (days)	22	18	28	23	19	28
$N_{-\text{bw}}$	3,255	2,593	4,060	3,410	2,593	4,060
$N_{+\text{bw}}$	3,095	2,581	3,754	3,243	2,581	3,754
N	21,106	21,106	21,106	21,106	21,106	21,106
	(b) SPECIAL NEEDS: DIAGNOSIS					
	SN diagnosis	Diagnosis before school	Diagnosis during school	Learning impairment	Behavioral problem	No diagnosis, dismissal
τ_{RD}	0.009	0.016	-0.006	-0.001	0.012	-0.012
	(0.031)	(0.015)	(0.027)	(0.025)	(0.017)	(0.016)
Bandwidth (days)	19	15	21	22	18	19
$N_{-\text{bw}}$	2,774	1,966	3,090	3,090	2,451	2,774
$N_{+\text{bw}}$	2,706	2,71	2,971	2,971	2,453	2,706
N	21,106	21,106	21,106	21,106	21,106	21,106
	(c) SPECIAL NEEDS: TYPE					
	Speech impediment	Speech imped. (excl. dys.)	Dyslexia/ dyscalculia	ADHD	Physical disability	Domestic violence
τ_{RD}	-0.027	-0.025	-0.010	-0.009	-0.012**	-0.001
	(0.020)	(0.018)	(0.017)	(0.012)	(0.005)	(0.003)
Bandwidth (days)	23	20	29	15	26	22
$N_{-\text{bw}}$	3,255	2,925	4,194	2,131	3,900	3,090
$N_{+\text{bw}}$	3,095	2,817	3,907	2,211	3,647	2,971
N	21,106	21,106	21,106	21,106	21,106	21,106

Notes: Estimates for τ_{RD} correspond to the treatment effect derived in section 3. All models include birth cohort specific effects and indicators for gender and non-native speaker. Heteroskedasticity-robust standard errors are reported in parentheses. *, **, and *** denote significance at the 10%, 5% and 1% level, respectively. Data are from the School Psychological Service St. Gallen, the Ministry of Education of the canton of St. Gallen, the Stellwerk test service provider, the Swiss Federal Statistical Office, and the Swiss Central Compensation Office.

Table A8: Donut Regression Excluding Seven Days Before and After the Cutoff

	(1)	(2)	(3)	(4)	(5)	(6)
	(a) Referral to the SPS					
	Referred to the SPS	No diagnosis, dismissal	Before school	During school	Learning impairment	Behavioral problem
τ_{RD}	-0.069***	-0.029***	0.003	-0.056***	-0.023	-0.025*
	(0.021)	(0.010)	(0.012)	(0.017)	(0.020)	(0.014)
Bandwidth (days)	43	47	45	60	42	40
$\bar{Y}_{-\text{bw}}$	0.341	0.054	0.071	0.270	0.233	0.082
$N_{-\text{bw}}$	6,138	6,718	6,416	8,686	6,138	5,719
$N_{+\text{bw}}$	6,164	6,825	6,495	8,921	6,164	5,690
N	49,907	49,907	49,907	49,907	49,907	49,907
	(b) Special needs: Diagnosis					
	SN diagnosis	Nr. of consultations	Diagnosis before school	Diagnosis during school	Learning impairment	Behavioral problem
τ_{RD}	-0.036*	-0.461*	0.017*	-0.045***	-0.016	-0.017
	(0.019)	(0.256)	(0.009)	(0.016)	(0.019)	(0.013)
Bandwidth (days)	52	41	50	65	41	42
$\bar{Y}_{-\text{bw}}$	0.287	2.670	0.044	0.243	0.203	0.070
$N_{-\text{bw}}$	7,577	5,855	7,320	9,345	5,855	5,998
$N_{+\text{bw}}$	7,784	5,850	7,449	9,595	5,850	6,015
N	49,907	49,907	49,907	49,907	49,907	49,907
	(c) Special needs: Type					
	Speech impediment	Speech imped. (excl. dys.)	Dyslexia/ dyscalculia	ADHD	Physical disability	Domestic violence
τ_{RD}	-0.019	-0.011	-0.021	-0.006	-0.001	0.001
	(0.016)	(0.012)	(0.015)	(0.007)	(0.003)	(0.003)
Bandwidth (days)	43	53	39	64	60	53
$\bar{Y}_{-\text{bw}}$	0.133	0.083	0.107	0.035	0.005	0.003
$N_{-\text{bw}}$	6,138	7,712	5,581	9,086	8,538	7,712
$N_{+\text{bw}}$	6,164	7,930	5,556	9,340	8,782	7,930
N	49,907	49,907	49,907	49,907	49,907	49,907

Notes: Estimates for τ_{RD} correspond to the treatment effect derived in section 3. All models include birth cohort specific effects and indicators for gender and non-native speaker. Heteroskedasticity-robust standard errors are reported in parentheses. *, **, and *** denote significance at the 10%, 5% and 1% level, respectively. Data are from the School Psychological Service St. Gallen, the Ministry of Education of the canton of St. Gallen, the Stellwerk test service provider, the Swiss Federal Statistical Office, and the Swiss Central Compensation Office.

Figure A1: Distribution of Birth Dates

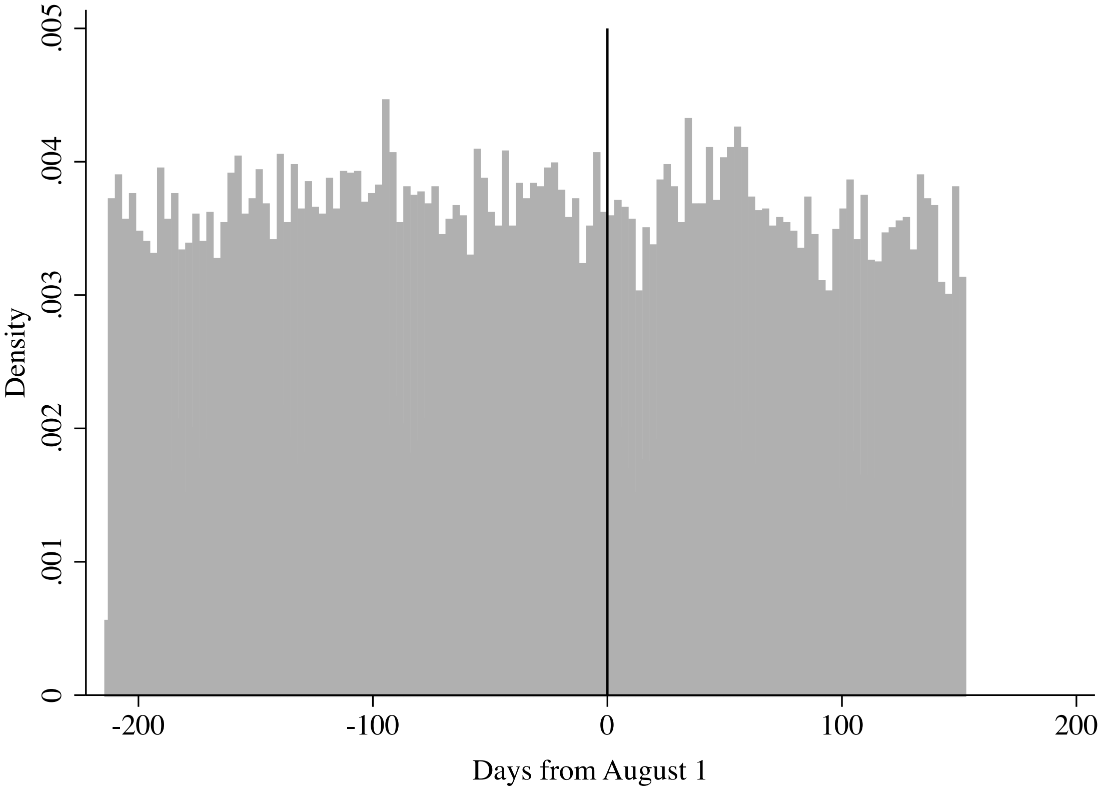

Notes: Data are from the Stellwerk test service provider.

Figure A2: Covariate Balance

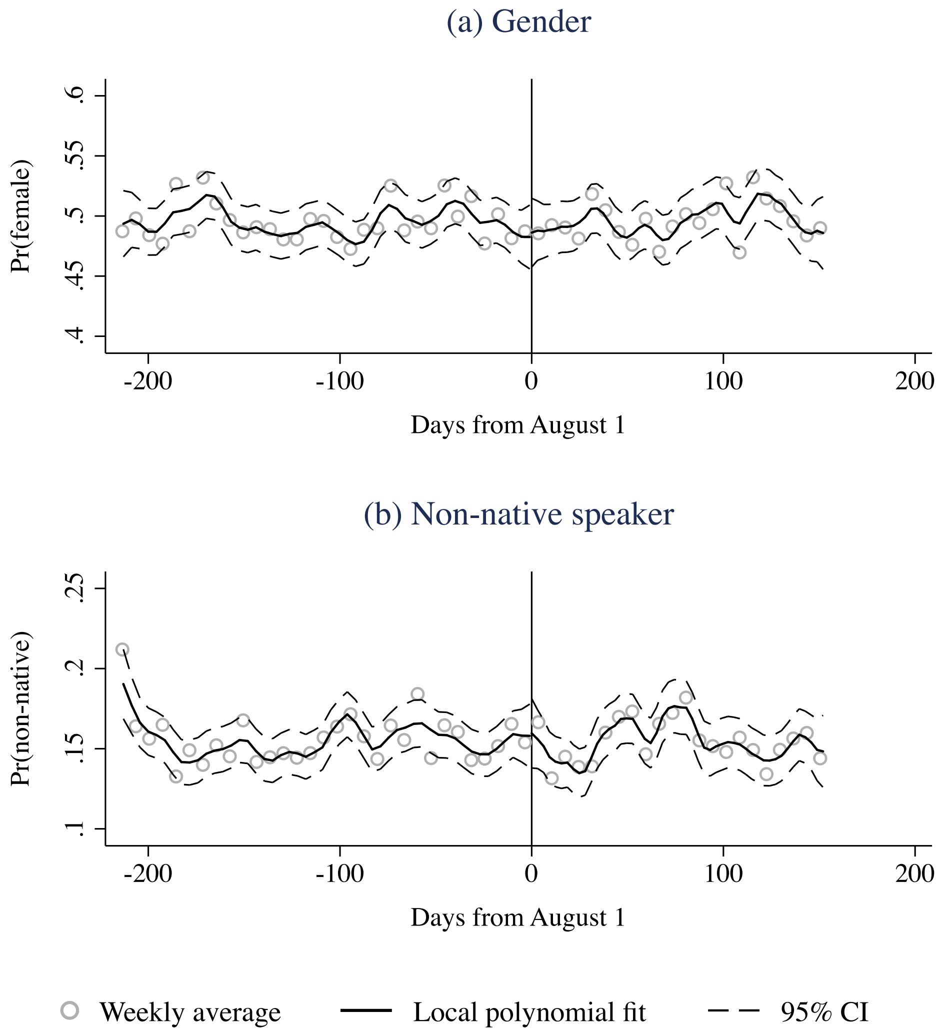

Notes: Data are from the Stellwerk test service provider.

Figure A3: Referrals to the SPS

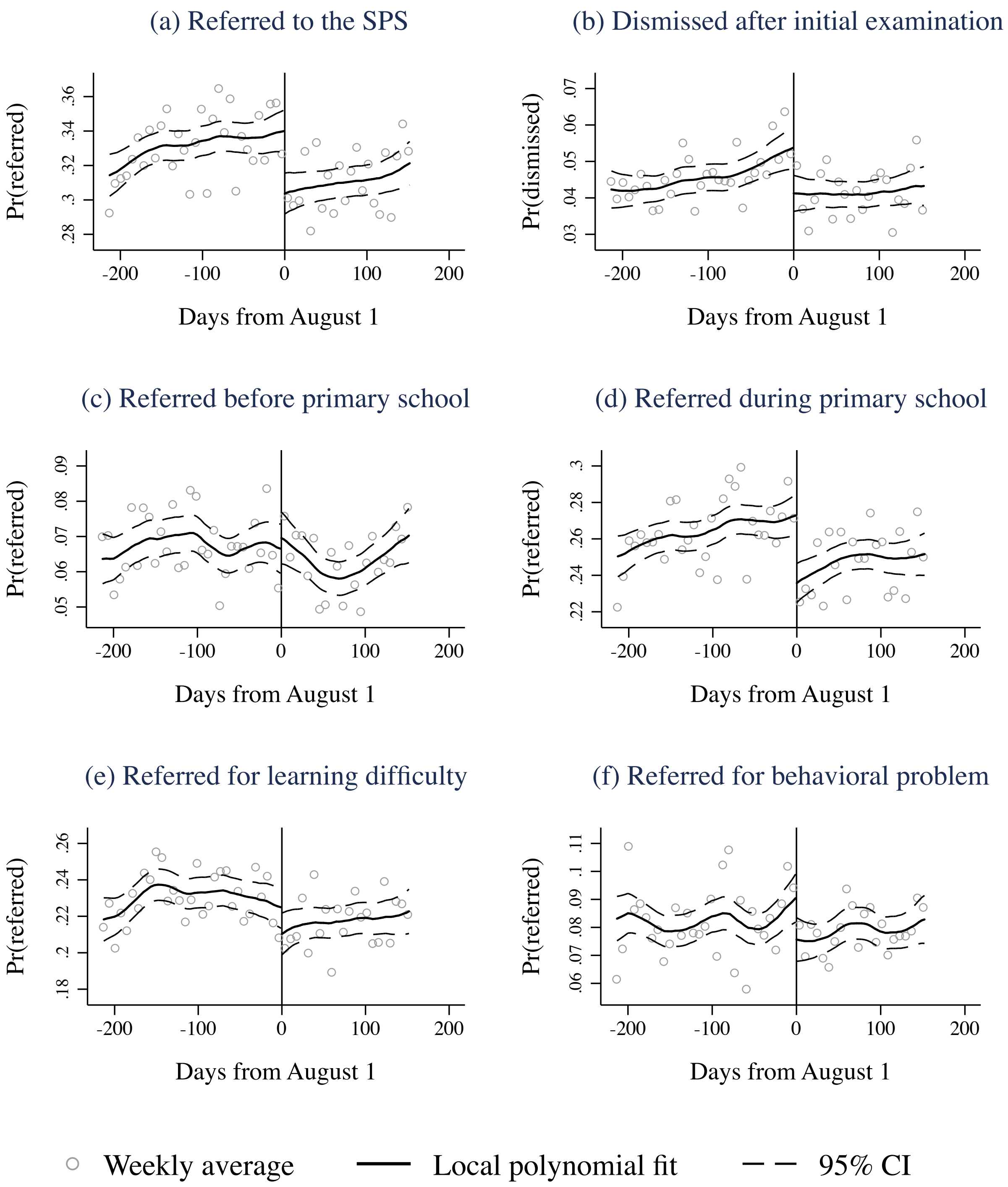

Notes: Discontinuity graphs plotting weekly averages and cutoff-dependent local linear regression estimates of school starting age on measures of special needs referrals. Local linear regression results are based on triangular kernel weights and a bandwidth choice following Calonico et al. (2018). Data are from the Stellwerk Test service provider and the School Psychological Service St. Gallen.

Figure A4: Special Needs Diagnoses, Timing, and Type

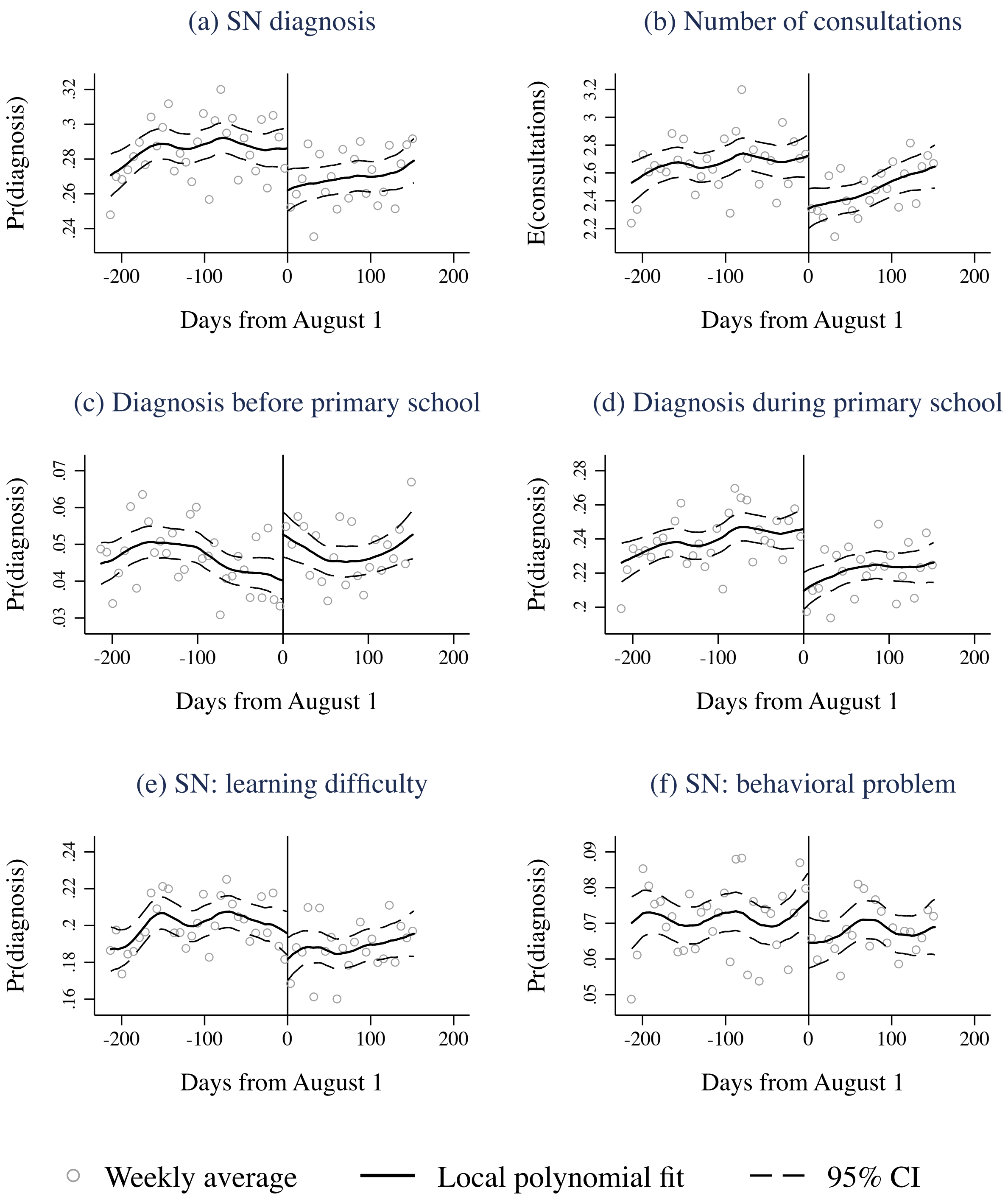

Notes: Discontinuity graphs plotting weekly averages and cutoff-dependent local linear regression estimates of school starting age on measures of special needs diagnoses. Local linear regression results are based on triangular kernel weights and a bandwidth choice following Calonico et al. (2018). Data are from the Stellwerk Test service provider and the School Psychological Service St. Gallen.

Figure A5: Special Needs Types

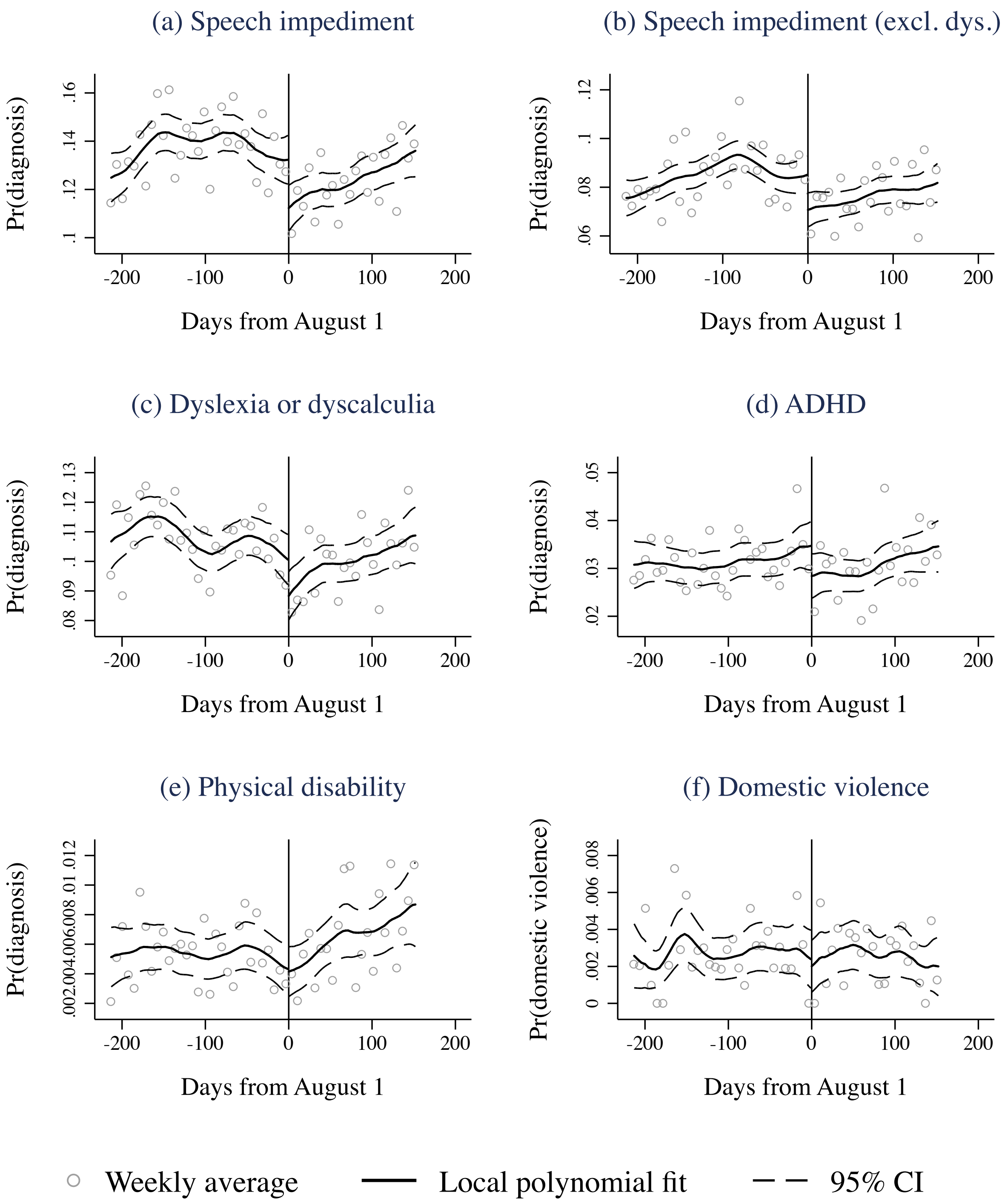

Notes: Discontinuity graphs plotting weekly averages and cutoff-dependent local linear regression estimates of school starting age on measures of special needs diagnoses. Local linear regression results are based on triangular kernel weights and a bandwidth choice following Calonico et al. (2018). Data are from the Stellwerk Test service provider and the School Psychological Service St. Gallen.

Figure A6: Graphical Analysis for Educational Outcomes

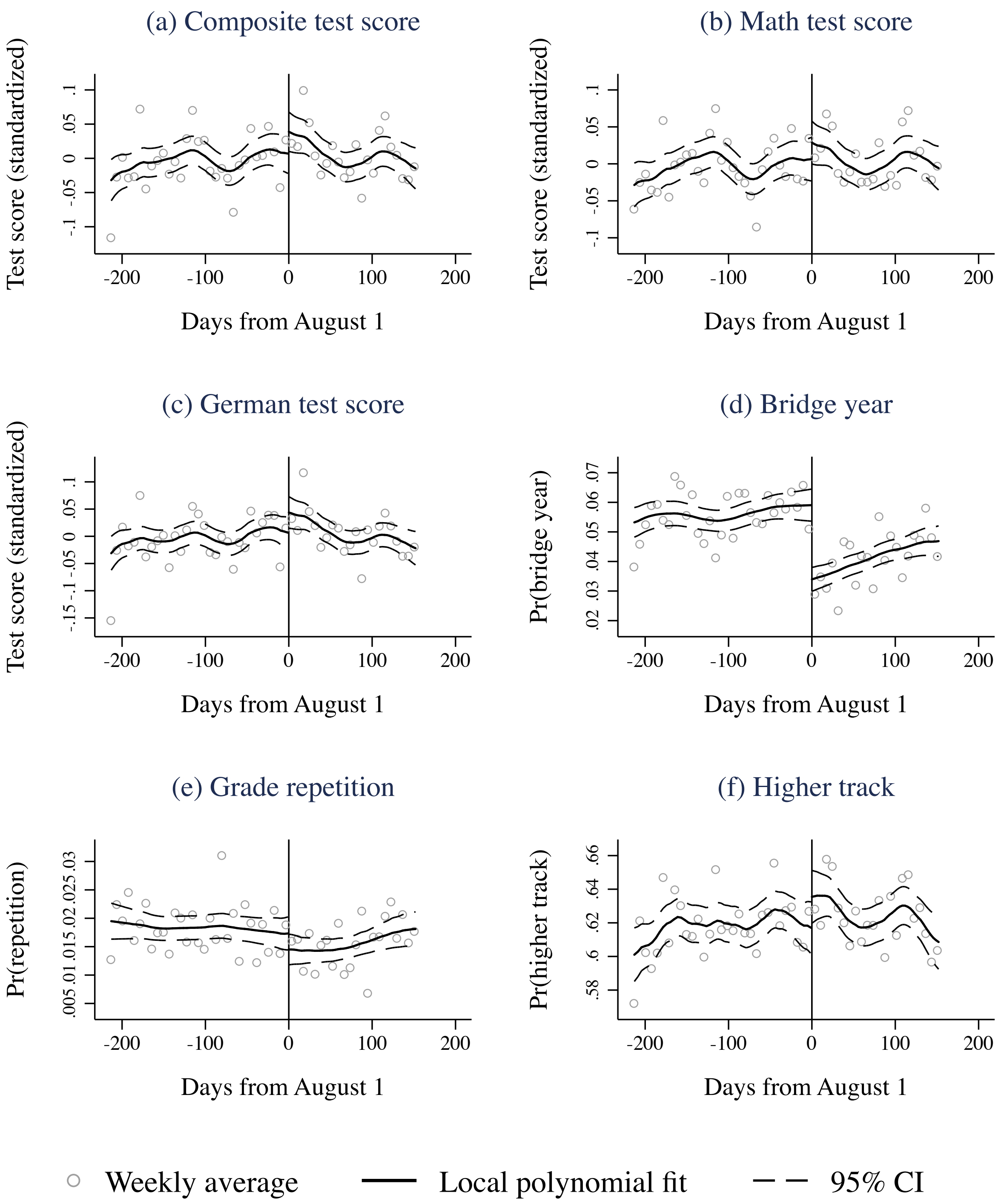

Notes: Data are from the School Psychological Service St. Gallen, the Ministry of Education of the canton of St. Gallen, and the Stellwerk test service provider.

Figure A7: Graphical Analysis for Education Trajectories

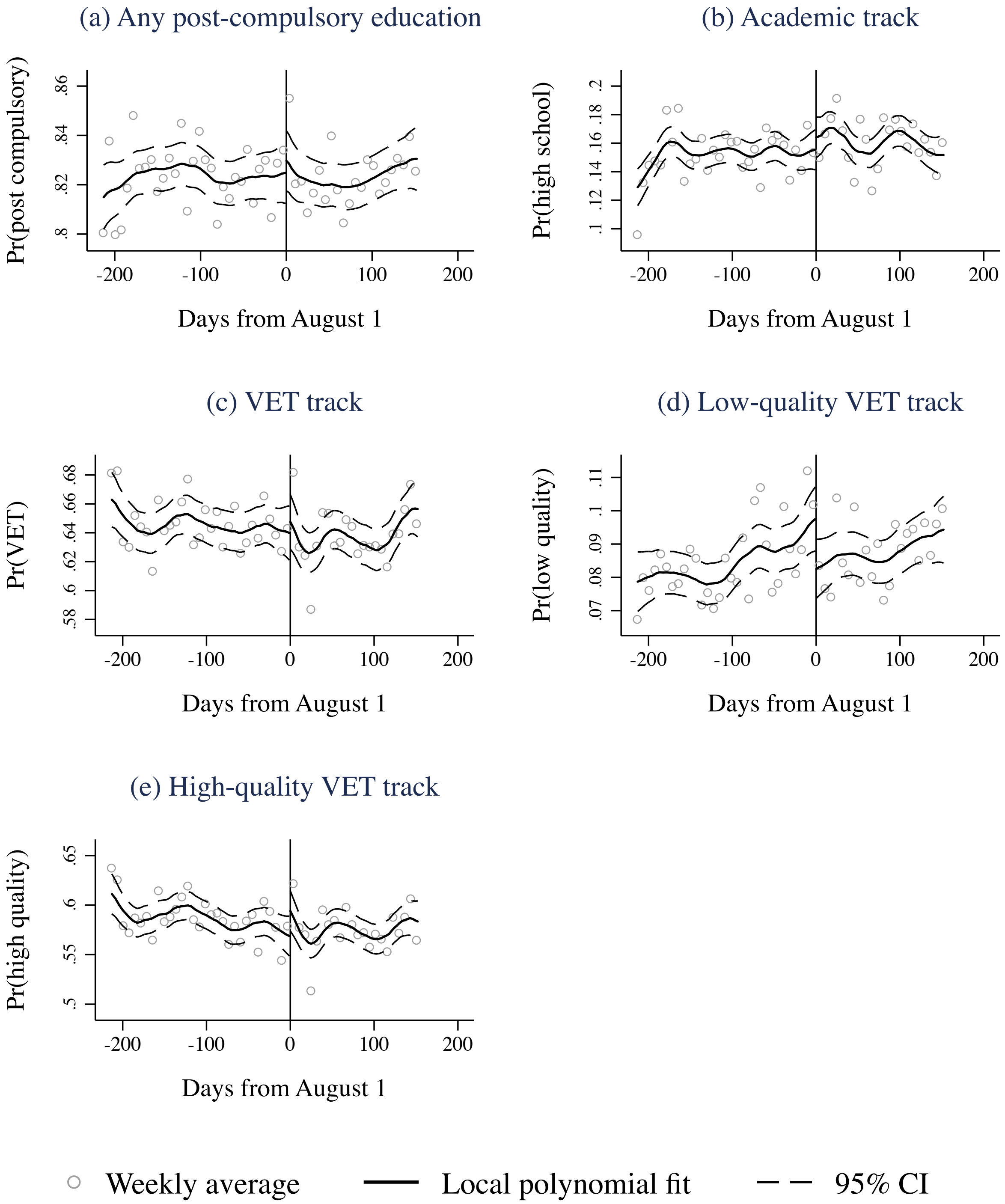

Notes: Data are from the School Psychological Service St. Gallen, the Ministry of Education of the canton of St. Gallen, and the Stellwerk test service provider.

Figure A8: First Stage Effect by Birth Year

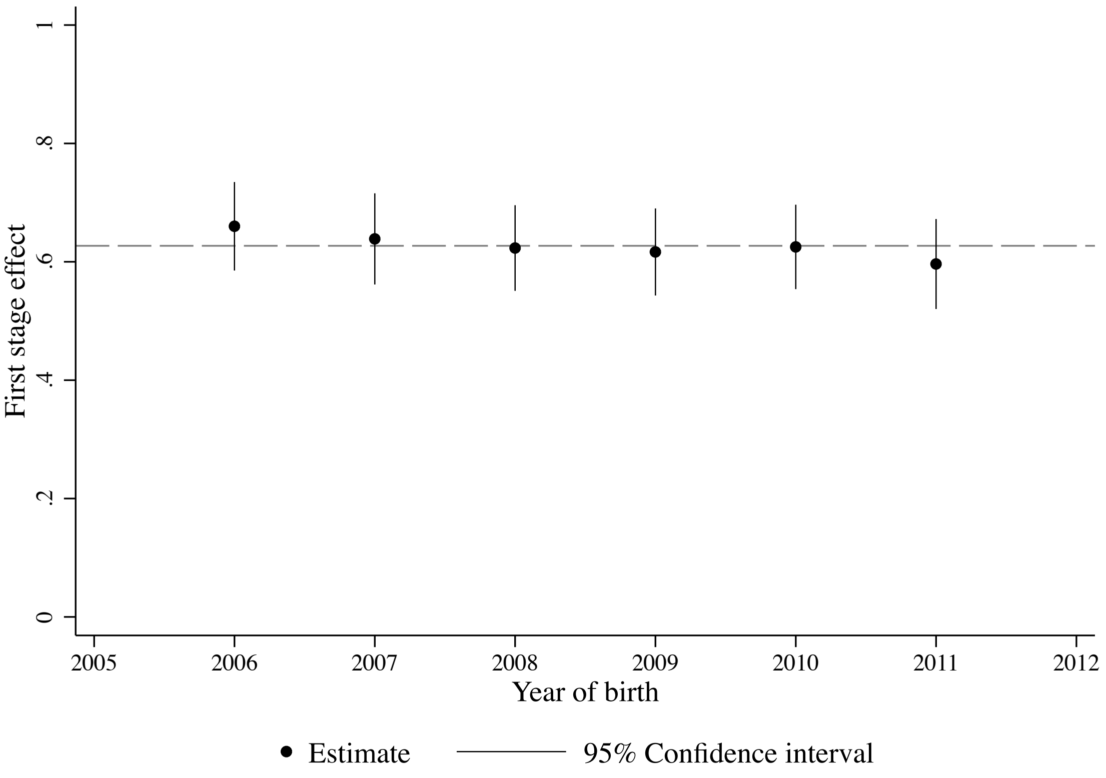

Notes: Local linear regression results are based on triangular kernel weights and a bandwidth choice following Calonico et al. (2018). Sample sizes are as follows: 4,474 for 2006, 4,616 for 2007, 4,817 for 2008, 4,926 for 2009, 4,926 for 2010, and 4,939 for 2011. The dashed line indicates the overall first stage effect. Data are from the Swiss Federal Statistical Office.

Figure A9: Bandwidth Variations

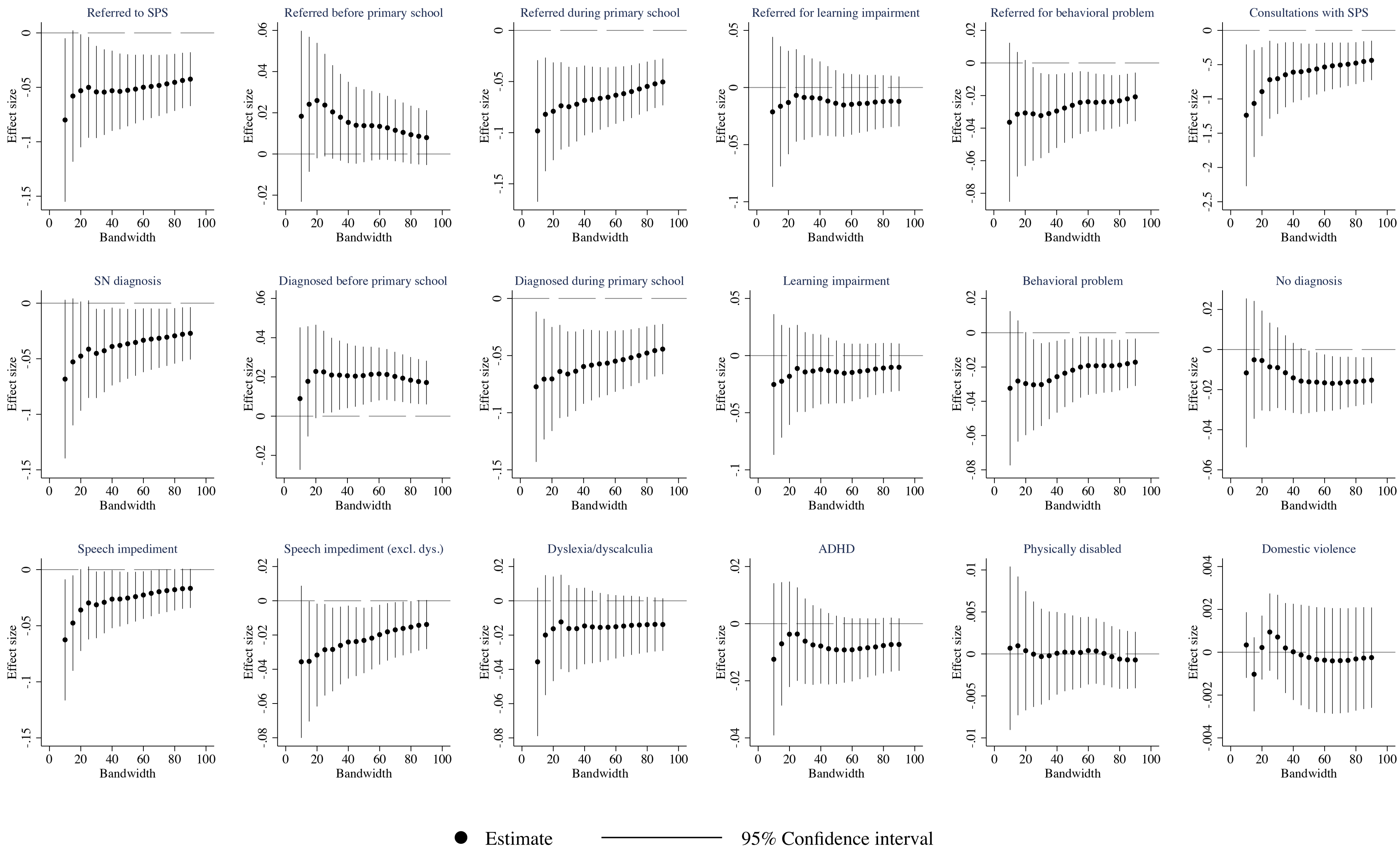

Notes: Data are from Stellwerk test service provider and the School Psychological Service St. Gallen.

Figure A10: Attrition Analysis

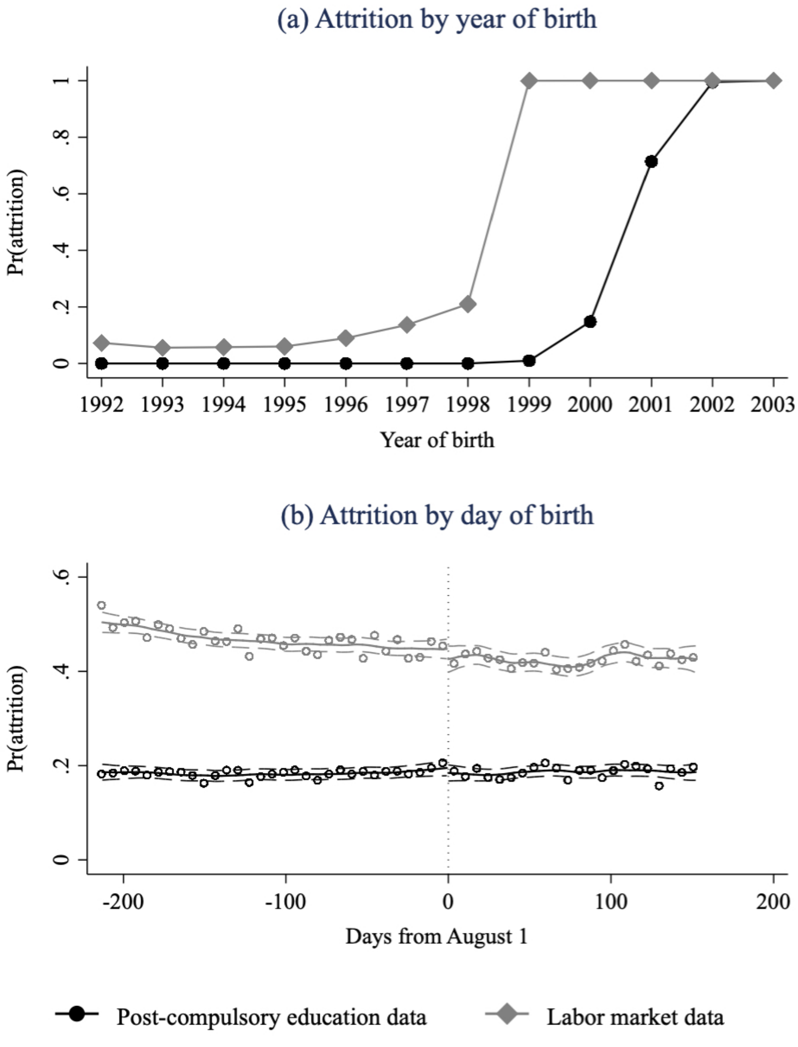

Notes: Data are from the School Psychological Service St. Gallen, the Ministry of Education of the canton of St. Gallen, and the Stellwerk test service provider.
